# miR-16 integrates signal pathways in myofibroblasts: determinant of cell fate necessary for fibrosis resolution

**DOI:** 10.1038/s41419-020-02832-z

**Published:** 2020-08-07

**Authors:** Qin Pan, Can-Jie Guo, Qing-Yang Xu, Jin-Zhi Wang, Han Li, Chun-Hua Fang

**Affiliations:** 1 Department of Gastroenterology, Xin-Hua Hospital, School of Medicine, Shanghai JiaoTong University, Shanghai, 200092 China; 2 Department of Gastroenterology, Ren-Ji Hospital, School of Medicine, Shanghai JiaoTong University, Shanghai, 200001 China; 3 School of Electronics and Information Engineering, Tong-Ji University, Shanghai, 201804 China

**Keywords:** Liver diseases, Liver fibrosis

## Abstract

Liver fibrosis is characterized by the transdifferentiation of hepatic stellate cells (HSCs) to myofibroblasts and poor response to treatment. This can be attributed to the myofibroblast-specific resistance to phenotype reversal. In this study, we complemented miR-16 into miR-16-deficient myofibroblasts and analyzed the global role of miR-16 using transcriptome profiling and generating a pathway-based action model underlying transcriptomic regulation. Phenotypic analysis of myofibroblasts and fibrogenic characterization were used to understand the effect of miR-16 on phenotypic remodeling of myofibroblasts. miR-16 expression altered the transcriptome of myofibroblasts to resemble that of HSCs. Simultaneous targeting of Smad2 and Wnt3a, etc. by miR-16 integrated signaling pathways of TGF-β and Wnt, etc., which underlay the comprehensive regulation of transcriptome. The synergistic effect of miR-16 on the signaling pathways abolished the phenotypic characteristics of myofibroblasts, including collagen production and inhibition of adipogenesis. In vivo, myofibroblast-specific expression of miR-16 not only eliminated mesenchymal cells with myofibroblast characteristics but also restored the phenotype of HSCs in perisinusoidal space. This phenotypic remodeling resolved liver fibrosis induced by chronic wound healing. Therefore, miR-16 may integrate signaling pathways crucial for the fate determination of myofibroblasts. Its global effect induces the reversal of HSC-to-myofibroblast transdifferentiation and, subsequently, the resolution of fibrogenesis. Taken together, these findings highlight the potential of miR-16 as a promising therapeutic target for liver fibrosis.

## Introduction

Liver fibrosis, a serious health problem worldwide, is induced by chronic liver injuries (hepatitis, alcoholism, cholestasis, etc.), which often lead to the development of hepatocellular carcinoma^1^. Liver fibrosis is characterized by the transdifferentiation of hepatic stellate cells (HSCs), a kind of adipogenic cells, toward myofibroblasts^2^. Multiple signaling pathways act in concert to enable this phenotypic transition^2,3^. Thus, such a complex network makes it difficult to target a single biomolecule, reverse the HSC-to-myofibroblast transdifferentiation, and ensure good response to treatment.

In contrast to most agents that act through single target, miRNA demonstrates a unique pattern, mainly multiple targeting, of gene regulation^4^. This pleiotropic effect highlights the importance of miRNAs in the phenotypic determination of various types of cells^5–9^. Among phenotype-regulating miRNAs, miR-16 is the most extensively explored one ever since its identification in 2005^10,11^, and serves as internal control of miRNA analysis for an universal and relative stable expression^12^. miR-16 undergoes loss of expression during the transdifferentiation of HSCs^13^. Its deficiency correlates to the myofibroblast-specific phenotype and apoptosis resistance, via Bcl-2-targeted activation of the mitochondrial apoptosis pathway^13^. Therefore, miR-16 is highlighted to be a potential in the phenotype controlling of myofibroblasts. However, whether miR-16 synergistically regulates multiple targets in a disease- or phenotype-specific manner and its association with the global regulation of mRNAs remain to be understood. The numerous targets of miR-16 had lent increasing ambiguity to its global effect, thereby making it difficult to understand the role of miR-16 in the resolution of fibrosis.

Muscle-specific miR-145 has been recently reported to function during the conversion of adult fibroblasts into smooth muscle cells with a contractile phenotype^6,7^. miR-206 (also muscle-specific) blocks the maturation of osteoblasts^8^ and growth of rhabdomyosarcoma by targeting myogenic differentiation^14^. Neurogenetic miRNAs, miR-124a and miR-125b, also help in the formation of mature neurons from the neuroblastoma line SH-SY5Y and neural progenitor ReNcell VM^9^. Thus, miRNAs function in a synergistic manner. miR-34c overexpression in HeLa cells upregulates germ cell-specific genes^15^. miR-1 alters the transcriptome of HeLa cells to resemble that of muscle cells^16^. Depletion of miR-23b during hepatocyte differentiation promotes the expression of bile duct-specific genes^17^. Therefore, transcriptome regulation may underlie the global action of miRNAs. Despite affecting multiple genes, miRNAs show limited base-pairing to the miRNA-regulated genes^18–21^. miRNAs possess preference for signaling pathway targets that highlights their importance as therapeutic targets^22–24^. Accordingly, we hypothesized that miRNAs exert a global cell-specific effect on the transcriptome and phenotype by integrated regulation of signaling pathways.

To test the synergistic actions of miR-16, we restored its expression in myofibroblasts and evaluated its impact on the cellular transcriptome. We used bioinformatic modeling to identify the central signaling pathways targeted to regulate the transcriptome. Wnt and transforming growth factor (TGF)-β signaling involved in adipogenesis and collagen production, respectively, revealed the role of miR-16 in myofibroblast transdifferentiation by integration of signaling pathways. The global effect of miR-16 on liver fibrosis was assessed on the basis of phenotype remodeling of myofibroblast.

## Materials and methods

### Isolation and identification of HSCs and myofibroblasts

HSCs were isolated from normal rats using in situ perfusion and density-gradient centrifugation^25^. Nevertheless, myofibroblasts were separated from rats with CCl_4_-induced liver fibrosis (Supplementary Fig. S1) by liver section and type IV collagenase/Pronase E/DNAse digestion. Freshly isolated myofibroblasts were purified using sequential centrifugation through 13% and 11% Nycodenz^26^. After 24 h culture for homogeneity, the viability (over 95%) and purity (up to 90%) of both HSCs and myofibroblasts were verified (Supplementary Fig. S2). Cell morphology and phenotype was assayed using oil red O staining and immunofluorescence for biomarkers (α-SMA and desmin) (Supplementary Fig. S2)^13,25,26^.

### Restoration of miR-16 levels in myofibroblasts

Rat HSCs and myofibroblasts were divided into seven groups at random, namely HSC, myofibroblast, pLV-GFP-treated myofibroblast, pLV-miR-16-treated myofibroblast, miR-16 inhibitor-treated myofibroblast, Smad2 small interfering RNA (siRNA)-treated myofibroblast, and Wnt3a siRNA-treated myofibroblast (*n* = 9 samples/group). The pLV-miR-16-treated and pLV-GFP-treated groups were infected with pLV-miR-16 (containing pre-rno-miR-16) and pLV-GFP (containing pre-rno-miR-16 with 5′-GGGGGG**-**3′ instead of the seed sequence), respectively, at 1.0 × 10^8^ transduction unit (TU)/ml and multiplicity of infection of 30. The group of miR-16 inhibitor-treated myofibroblast was administrated by both 20 µM miR-16 inhibitor (GenePharm, Shanghai, China) and pLV-miR-16 as mentioned above. Whereas Smad2 siRNA (GenePharm, Shanghai, China), and Wnt3a siRNA (GenePharm, Shanghai, China) were transfected into groups of Smad2 siRNA-treated myofibroblast and Wnt3a siRNA-treated myofibroblast, respectively, according to the manufacturer’s instructions.

Total RNA was extracted from cells 24 h after isolation (HSC group and myofibroblast group of rats, HSC, and myofibroblast of human) or 48 h post transduction (pLV-miR-16-treated myofibroblast group, pLV-GFP-treated myofibroblast group). Thereafter, we performed stem-loop quantitative reverse transcription-PCR (RT-QPCR) for miR-16 and miR-15b of the miR-16 cluster using the TaqMan MicroRNA Assay (Applied Biosystems, Foster City, CA, USA). miRNA expression was normalized to U87 snRNA levels.

### Microarray analysis and bioinformatic modeling of miR-16

Transcriptome profiling was performed using Affymetrix rat 230 2.0 arrays (Affymetrix, Santa Clara, CA, USA) 24 h after cell isolation (groups of HSC and myofibroblast) or 12–48 h after lentiviral transfection (groups of pLV-miR-16-treated and pLV-GFP-treated myofibroblast).

The predicted target gene sets of miR-16 were obtained from databases of miRBase and TargetScan 5.1, and intersected with miR-16-regulated gene set. These gene sets, after being supplemented with proven targets, were mapped to DAVID database to generate sets of signaling pathways^27^. The intersection between three sets of signaling pathways have been presented. Furthermore, signaling pathways relevant to miR-16 were filtered from the intersection upon enrichment against the background. Their roles and relationships were measured by biological-process-based categorization and generation of pathway–pathway interaction network, according to the annotations of the Kyoto Encyclopedia of Genes and Genomes (KEGG) and directed graph theory topology. Mediating the effect of most other signaling pathways (>3), 20% signaling pathways were classified as the critical nodes of pathway–pathway interaction network and the key executors of miR-16’s actions.

### Effect of miR-16 on targets within signaling pathways

We performed luciferase assays to determine the targeted effect of miR-16 on *SMAD2* and *Wnt3a*. Transcription of the targets (genes involved in signaling pathways and their downstream genes) of miR-16 were subsequently detected in seven groups of HSCs and myofibroblasts using RT-QPCR (Supplementary Table S1). The in vivo and in vitro expression of the targets were analyzed using immunofluorescence and quantified of western blotting.

### Functional analysis of myofibroblasts upon miR-16 restoration

Enzyme-linked immunosorbent assays (Cusabio, College Park, MD, USA) were used to determine the levels of collagen type I and III in the culture supernatants of seven groups of HSCs and myofibroblasts. Cell cycle progression and proliferation were assayed using propidium iodide staining and the Cell Counting Kit 8 (Dojindo, Kumamoto, Japan), respectively, after serum starvation for 24 h.

Moreover, the adipogenic activity of the seven groups of HSCs and myofibroblasts were assayed based on the expression of adipogenic transcription factors (TFs; PPARγ, C/EBPα, and RXRα) and normalized using β-catenin levels.

### Phenotypic characterization of myofibroblasts after targeted miR-16 normalization

Rats were randomized into the normal control group (*n* = 6), fibrosis model group (*n* = 5), pLV-miR-16-treated group (*n* = 5), and pLV-GFP-treated group (*n* = 5). After the 40% CCl_4_ administration (0.3 ml/kg) for 4 weeks, pLV-miR-16 (pLV-miR-16-treated group, 1.0 × 10^8^ TU/week) with α-SMA promoter^28^ or pLV-GFP (pLV-GFP-treated group, 1.2 × 10^8^ TU/week) was delivered, respectively, in vivo by superior mesentery vein catheterization for another 4 weeks^29^. The liver-targeted lentiviral transfection, with or without miR-16, was assessed by monitoring the green fluorescent protein (GFP) signal.

At the end of 8 weeks, the presence of dual-labeled Wnt3a/α-SMA and Smad2/α-SMA mesenchymal cells were monitored in liver sections by immunofluorescence. Transmission electron microscopy was used to identify ultrastructures specific to HSCs and myofibroblasts. Apoptosis and proliferation were assayed using terminal deoxynucleotidyl transferase-mediated dUTP nick end labeling (TUNEL) and immunohistochemical labeling of proliferating cell nuclear antigen (PCNA), respectively.

### Global effect of miR-16 on liver fibrosis

Hepatic histopathology of normal control, fibrosis model, pLV-miR-16-treated, and pLV-GFP-treated groups was assessed by hematoxylin and eosin staining and Sirius red staining. Collagen deposition was assayed using immunofluorescence and quantified by western blotting for collagen type I and III. Fibrosis staging was independently determined, in accordance with the Ishak staging system, by two pathologists who were not aware of the experiments. The protocols in study were approved by the Ethical Committee at Xinhua Hospital. Rats received humane care in accordance with the US Public Health Service Policy on Humane Care and Use of Laboratory Animals.

### Statistical analysis

Data have been represented as mean ± SD. Independent *t*-test and analysis of variance were applied for pair comparisons and multiple comparisons, respectively. A *χ*^2^-test was done with quadruple tabular form. Sample randomness was determined using the equal probability sampling method (SPSS 15.0). Differences with *P* < 0.05 were considered statistically significant.

## Results

### Restoration of miR-16 shaped the transcriptome of myofibroblast towards HSC-like one

As compared to that in HSCs, we observed significant downregulation of miR-16 in the myofibroblasts (Supplementary Fig. S3). Rodent myofibroblasts were then transduced with pLV-miR-16 at an efficiency of over 90% (Supplementary Figs. S4 and S5) after their isolation, 24 h cultural homogeneity, and identification. Although few changes in the transcriptome were observed at the 12 and 24 h time points, the up- and downregulated transcripts reached 1162 (3.74%) and 920 (2.96%), respectively, at 48 h with the restoration of miR-16 level (Supplementary Fig. S6). miR-15b levels, another member of the miR-16 cluster, remained constant during the same period (Supplementary Fig. S6).

**Fig. 1:**
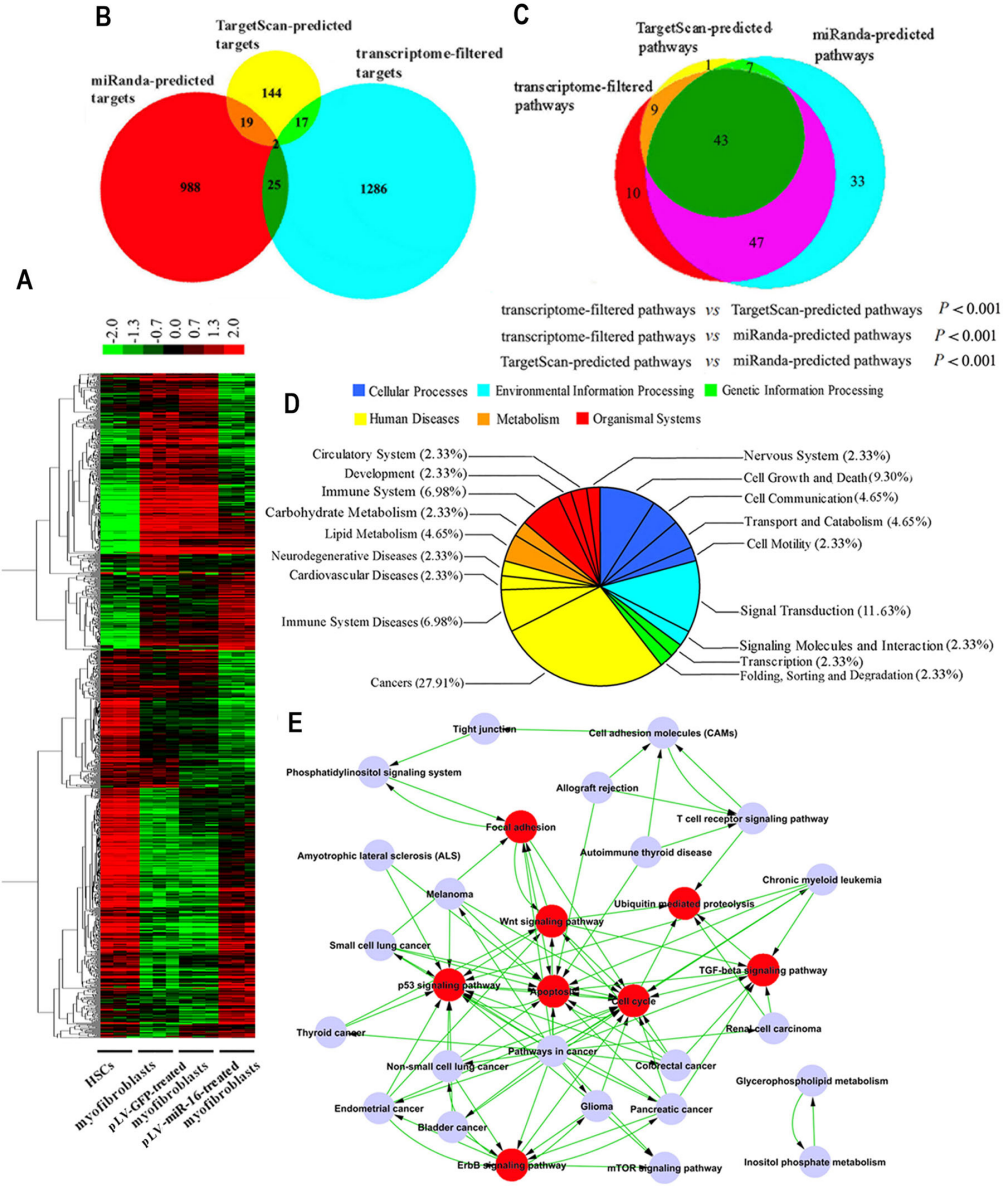
miR-16 induces the transcriptome of myofibroblasts toward that of hepatic stellate cells (HSCs) in an action model of signaling pathway integration. **a** Cluster heat map for the transcriptome of HSC, myofibroblast, and pLV-GFP-treated and pLV-miR-16-treated myofibroblast show the significantly downregulated (green) and upregulated (red) mRNAs specific to miR-16 loss (myofibroblast, pLV-GFP-treated myofibroblast) and restoration (pLV-miR-16-treated myofibroblast 48 h post transduction) (*n* = 3 per group). **b** There is limited intersection among miR-16-regulated genes and miR-16 targets predicted by miRanda or TargetScan algorithm, respectively. **c** Large intersection characterizes the signaling pathway sets (miRanda-predicted pathways, TargetScan-predicted pathways, and transcriptome-filtered pathways), which are predicted or tested to be under the control of miR-16, suggesting the signaling pathway-based effect of miR-16 that shapes the fibroblastic transcriptome of myofibroblasts toward the adipocyte-like one of HSCs. Equal probability sampling method evaluates the probability that the intersection of signaling pathway sets is obtained by random sampling. **d** Functional category of the intersected signaling pathways uncovers the global role of miR-16 that covers most of its recognized functions. **e** Pathway–pathway interaction network identifies the action model of miR-16 through integration of critical signaling pathways (red).

After validating the alterations in the transcriptome (Supplementary Fig. S7), the filtered genes were categorized by hierarchical clustering (Fig. 1a) and function (Supplementary Table S2). (1) Adipogenic genes, which characterizes HSCs, were increased after the administration of miR-16. (2) miR-16-regulated cytokines, collagens, and matrix metallopeptidases showed a rebalancing of extracellular matrix (ECM) production and zymohydrolysis, thereby inactivating the myofibroblasts and facilitating the regression of hepatic fibrosis. (3) Genes associated with cell cycle arrest in myofibroblasts were induced upon restoration of miR-16 expression, including the upregulation of cell cycle inhibitors and downregulation of cell cycle inducers. (4) A significant population of the upregulated transcripts participated in mitochondrial apoptosis; this could result in reduced apoptosis in myofibroblasts. In summary, the induction of miR-16 expression reversed the mRNA profile of myofibroblasts to that of HSCs.

### Bioinformatic modeling identified the targeted effect of miR-16 on signaling pathways

Using the miRanda, TargetScan algorithm, and transcriptomic filtration, three sets of gene targets (miRanda-predicted targets, TargetScan-predicted targets, and transcriptome-filtered targets) were generated, which corresponded to the transcripts predicted or tested to be regulated by miR-16. The intersection between these three sets, however, comprised a limited number of genes (Fig. 1b). Notably, the three sets of signaling pathways (miRanda-predicted pathways, TargetScan-predicted pathways, and transcriptome-filtered pathways), predicted or validated to be regulated by miR-16, significantly overlapped with each other (Fig. 1c, *P* < 0.001).

Using Gene Ontology, we functionally annotated the miR-16-regulated genes (Supplementary Table S3). This included a major proportion of intracellular signaling cascades (7.04%, *P* = 0.001). In addition, 32.3% of the miR-16-regulated genes were annotated to be members of signaling pathways, significantly higher than the percentage of background (*P* < 0.001) (Supplementary Fig. S8). The integrated effect of a specific set of signaling pathways, therefore, seems to underlie the impact of miR-16 on the transcriptome.

Organization of the signaling pathways into functional categories revealed the synergistic effect of 43 intersected signaling pathways on the basis of KEGG annotation. Proportionally, Cancers, Signal Transduction, Cell Growth and Death, etc. were the most represented categories (Fig. 1d). Among these intersected pathways, eight signaling pathways were the most crucial nodes: Cell cycle, Apoptosis, TGF-β signaling pathway, Wnt signaling pathway, p53 signaling pathway, Focal adhesion, Ubiquitin-mediated Proteolysis, and ErbB signaling pathway, as they served as the nodes of the pathway–pathway interaction network (Fig. 1e).

### miR-16 inhibited the fibrotic and proliferative properties of myofibroblasts by targeting TGF-β signaling pathway

**Fig. 2:**
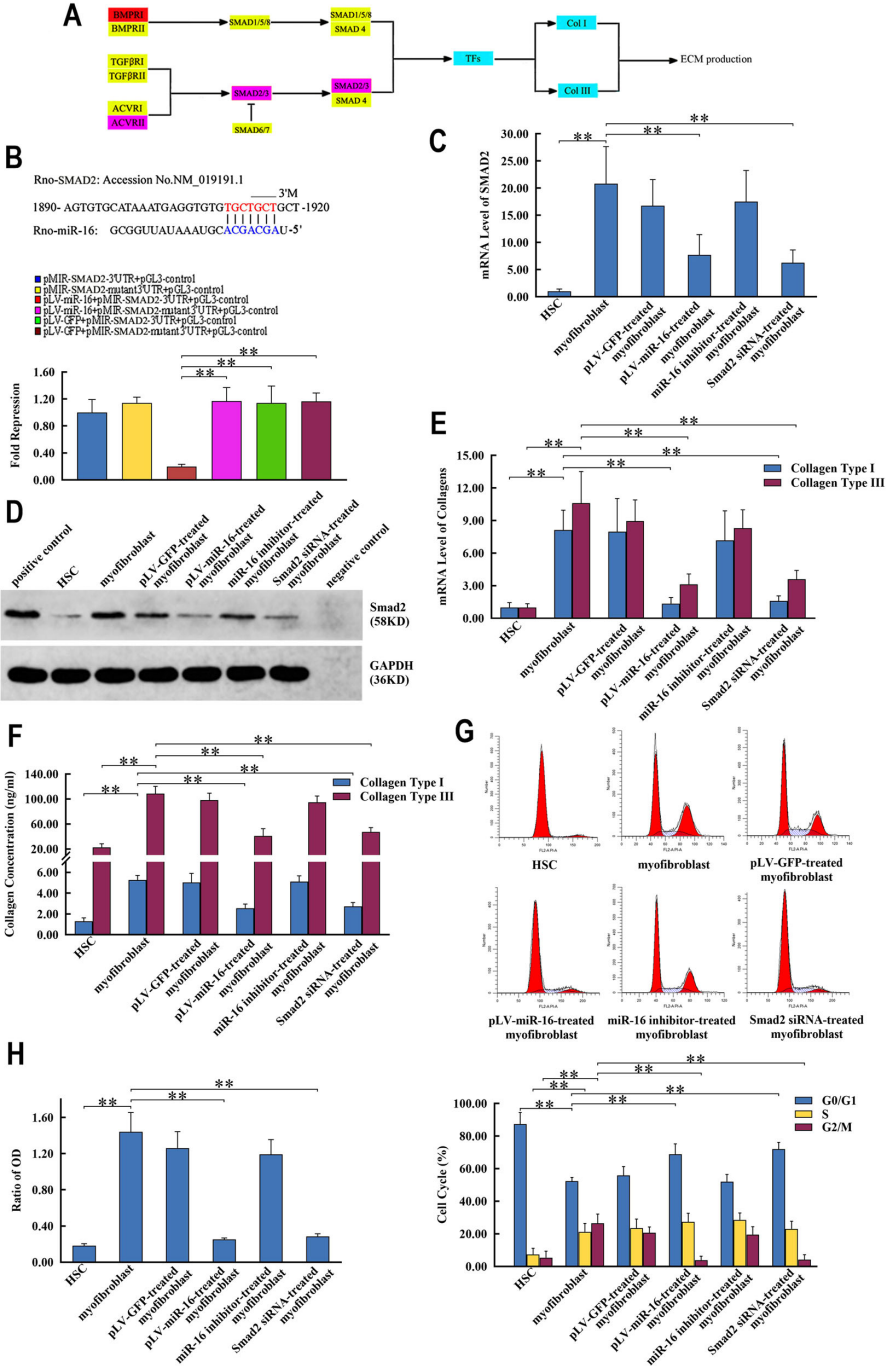
miR-16 abrogates fibrosis-inducing characteristics of myofibroblasts by TGF-β signaling pathway. **a** Schematic representation of miR-16’s effect on TGF-β signaling pathway. Red, blue, and violet ellipses represent genes affected by miR-16 predictably, transcriptionally, and both. **b** Complementary binding of SMAD2 mRNA and “seed region” of miR-16 within 3′-untranslated region (3′-UTR, upper panel). Dual luciferase assay confirmed the target effect of miR-16 on SMAD2, which could be diminished through base replacement (bottom panel). **c**, **d** Complementation between miR-16 and target mRNA sequence downregulates the transcription (**c**) and translation (**d**) levels of SMAD2 in pLV-miR-16-treated myofibroblast. **e**, **f** Effect of miR-16 on SMAD2 inhibits the transcription of *COLIA*, *COLIIIA1* (**e**) and secretion of collagen type I, III (**f**) via the TGF-β signaling pathway. **g**, **h** miR-16 blocks the cell cycle (**g**) and proliferation (**h**) of myofibroblasts via the TGF-β signaling pathway. The effects of miR-16 on SMAD2 and fibrosis-inducing characteristics of myofibroblasts via the TGF-β signaling pathway are mimicked by SMAD2-specific RNAi and abolished by miR-16 inhibitor. Values are expressed as means ± SD. *P < 0.05, **P < 0.01.

Dual luciferase assays showed that miR-16 targeted SMAD2, a key member of the TGF-β signaling pathway (Fig. 2a, b). Protein and mRNA levels of SMAD2 decreased with the expression of miR-16 (Fig. 2c, d and Supplementary Fig. S9A). The mRNA levels of genes downstream to the TGF-β signaling pathway (collagen type I and III) also decreased (Fig. 2e). miR-16 reduced the concentrations of collagen type I and III in the culture supernatant, indicating net changes in ECM synthesis and secretion (Fig. 2f). Inactivation of TGF-β signaling pathway also induced the G_0_/G_1_ block and reduced G_2_/M proportion (Fig. 2g) of myofibroblasts, resulting in cell cycle arrest and inhibition of proliferation (Fig. 2h). Dramatically, the effects of miR-16 on ECM production and myofibroblasts proliferation were recapitulated by SMAD2-specific RNA interference and abrogated by miR-16 inhibitor (Fig. 2c–h and Supplementary Fig. S9A).

### miR-16 restored adipogenic characteristics of myofibroblasts by targeting Wnt signaling pathway

**Fig. 3:**
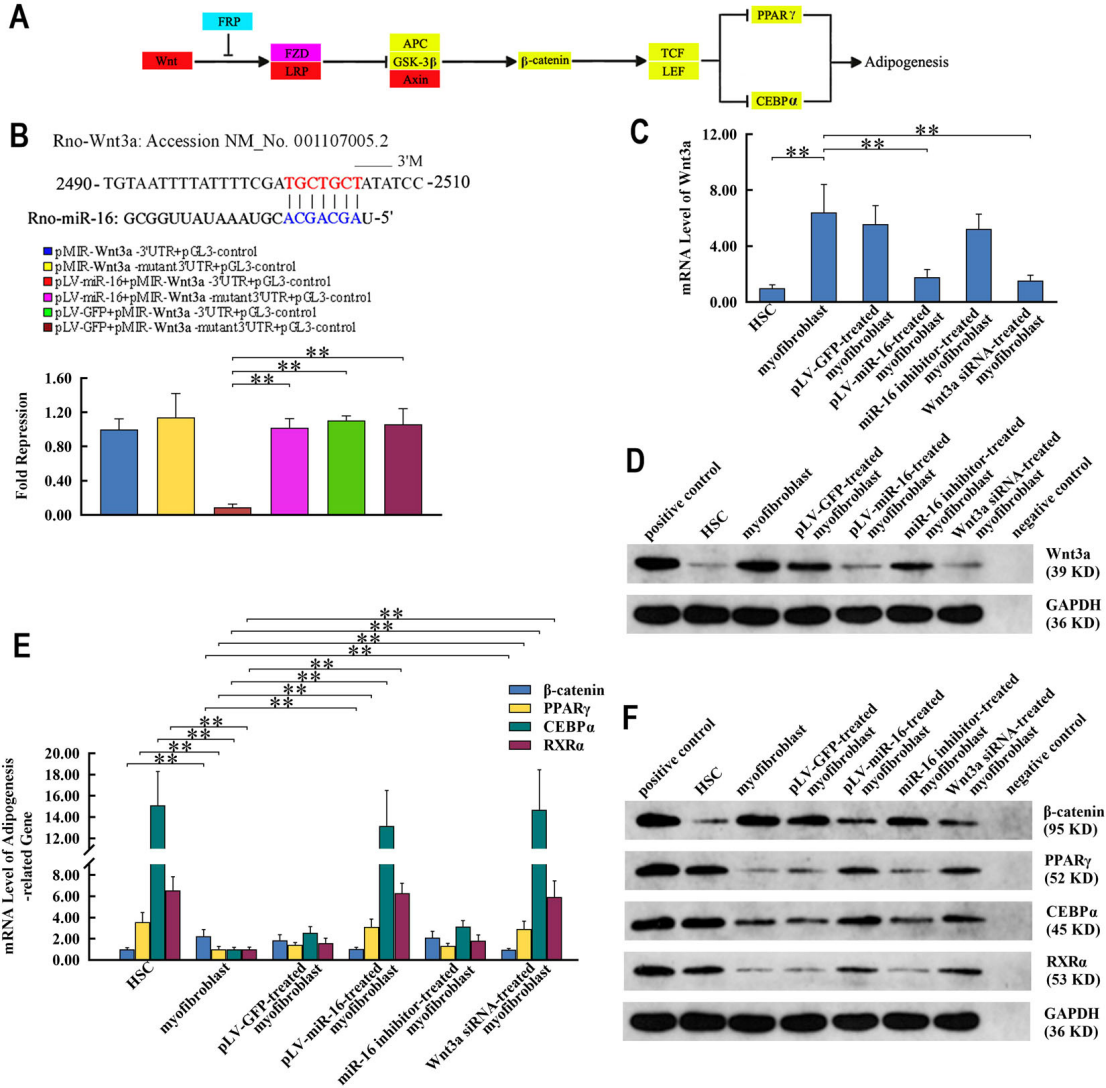
miR-16 activates adipogenic characteristics of myofibroblasts by signaling pathway of Wnt. **a** Schematic representation of miR-16’s effect on Wnt signaling pathway. Red, blue, and violet ellipses represent genes affected by miR-16 predictably, transcriptionally, and both. **b** Wnt3a binds to the “seed region” of miR-16 via complementary sequence within the 3′-UTR (upper panel). Dual luciferase assay verified the targeted effect of miR-16 on Wnt3a (bottom panel). **c**, **d** Complementation between miR-16 and Wnt3a decreased its expression at the levels of transcription (**c**) and translation (**d**) in pLV-miR-16-treated myofibroblast. **e**, **f** Effect of miR-16 on Wnt3a upregulates the transcription (**e**) and translation (**f**) levels of C/EBPα, PPARγ, and RXRα by blocking the β-catenin-mediated Wnt signaling pathway. The effects of miR-16 on Wnt3a and adipogenic characteristics of myofibroblasts via the Wnt signaling pathway are mimicked by Wnt3a-specific RNAi and abolished by miR-16 inhibitor. Values are expressed as means ± SD. *P < 0.05, **P < 0.01.

Wnt3a, a component of Wnt signaling, was another target of miR-16 (Fig. 3a, b). Wnt3a was transcriptionally and translationally downregulated in pLV-miR-16-treated myofibroblasts (Fig. 3c, d and Supplementary Fig. S9B). This resulted in the reduced accumulation of β-catenin, thereby promoting the mesenchymal phenotype in myofibroblasts (Fig. 3e, f and Supplementary Fig. S10). On the contrary, an elevated expression of adipogenic TFs (C/EBPα, PPARγ, and RXRα) took place simultaneously with statistical significance (Fig. 3e, f and Supplementary Fig. S10). Moreover, depletion of Wnt3a in the myofibroblasts regulated the expression of mesenchymal markers and adipogenic TFs, mimicking those in miR-16-restored myofibroblasts (Fig. 3c–f and Supplementary Figs. S9B and S10). The miR-16 inhibitor reversed the changes in expression induced by miR-16 (Fig. 3c–f and Supplementary Figs. S9B and S10).

### miR-16 induced phenotype reversal from myofibroblast to HSC-like cell by integrated effects on signaling pathways

**Fig. 4:**
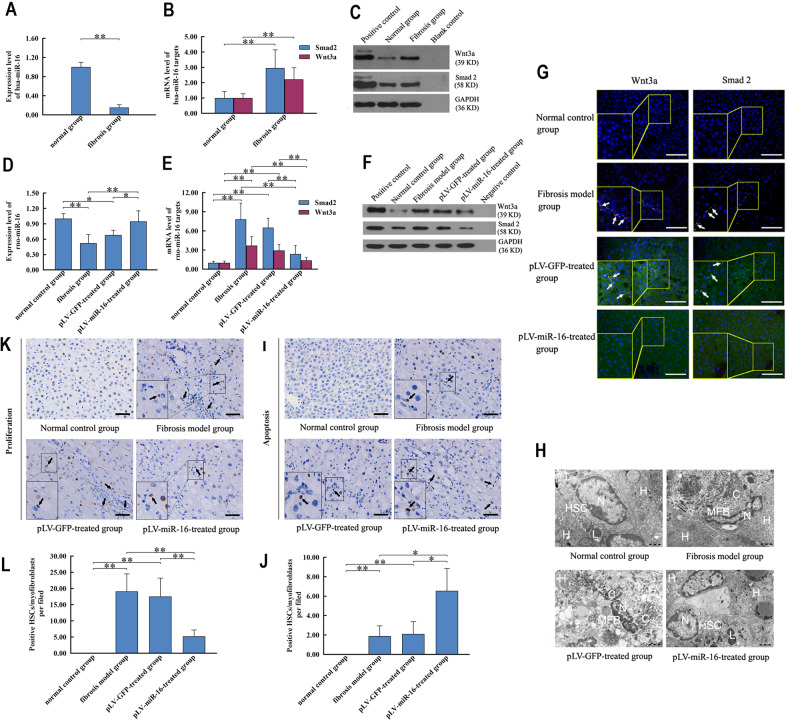
Synergistic effect of miR-16 results in the phenotypic reversal from myofibroblasts to HSC-like cells. **a**–**c** miR-16 deficiency (**a**) is accompanied by the increased expression of Smad2 and Wnt3a, at both transcription (**b**) and translation levels (**c**), in myofibroblasts of patients with liver fibrosis. **d**–**f** Effect of cell-specific restoration of miR-16 is characterized by miR-16 expression (**d**) and the level of its targets (**e**, **f**) in HSCs of normal control group and myofibroblasts of fibrosis model, pLV-GFP-treated, and pLV-miR-16-treated groups, respectively. **g** Colocalization of Smad2/α-SMA and Wnt3a/α-SMA, respectively, highlights the phenotypic loss of myofibroblast after miR-16 restoration. Left bottom panels show the enlarged image for rectangular fields. Blue, red, and green signals in cells represent the nucleus, Smad2/Wnt3a, and α-SMA (marker of myofibroblasts), respectively (scale bar: 100 μm). White asterisks indicate the myofibroblasts dual-positive for Smad2/α-SMA or Wnt3a/α-SMA. **h** Synergistic effect of miR-16 abolishes ultrastructural characteristics of myofibroblasts (i.e., spindle morphology, and intracellular and extracellular deposition of collagen filament), whereas restores those of HSCs (i.e., specific location in the perisinusoidal space, intracellular lipid droplets with high electronic density) (scale bar: 2000 nm). C, collagen fiber; H, hepatocyte; HSC, hepatic stellate cell; L, lipid droplet; MFB, myofibroblast; N, nuclei. **i**, **j** Terminal dexynucleotidyl transferase (TdT)-mediated dUTP nick end labeling (TUNEL) (**i**) and quantitative results (**j**) reveal the effect of miR-16 on apoptosis in HSCs of normal control group, and myofibroblasts of fibrosis model, pLV-GFP-treated, and pLV-miR-16-treated groups, respectively (scale bar: 20 μm). Asterisks indicate the positive cells. **k**, **l** Immunohistochemical staining of proliferating cell nuclear antigen (PCNA) (**k**) and its quantification (**l**) reveal the effect of miR-16 on proliferation in HSCs and myofibroblasts of four groups (scale bar: 20 μm). Asterisks indicate the positive cells. Values are expressed as means ± SD. *P < 0.05, **P < 0.01.

miR-16 was downregulated in myofibroblasts as compared to that in HSCs derived from rats and patients with liver fibrosis (Fig. 4a, d). We also observed an increase in the mRNA and protein levels of Smad2 and Wnt3a (Figs. 4b, c, e, f and Supplementary Figs. S11 and S12). In contrast, myofibroblast-targeted in vivo miR-16 restoration (Supplementary Fig. S13), performing from week 5 to 8, mitigated the miR-16 lacking (fibrosis model group vs. pLV-miR-16-treated group, *P* < 0.01) and the overexpression of its targets (Fig. 4d–f and Supplementary Fig. S12).

The targeting effect of miR-16 on Smad2 and Wnt3a reduced mesenchymal cells dual labeling for Smad2/α-SMA and Wnt3a/α-SMA (Fig. 4g and Supplementary Fig. S14). Ultrastructural findings, especially the existence of lipid droplets instead of the deposition of collagen filament, and regaining the spherical-like instead of stretched morphology, indicated that miR-16 shaped myofibroblasts towards the phenotype of HSCs (Fig. 4h). Despite the chemotactic stimuli, miR-16-induced HSC-like cells were localized to the sinusoidal space without aggregation at the site of the injury.

Analysis of apoptosis and proliferation reflected additional effects of miR-16 on phenotypic modulation. By TUNEL, an augment of positive signals enriching these HSC-like cells were shown in the pLV-miR-16-treated group rather than fibrosis model group and pLV-GFP-treated group (Fig. 4i, j). However, the pLV-miR-16-treated group exhibited the reduced level of proliferative marker (PCNA) in comparison to other two groups (Fig. 4k, l).

### miR-16-mediated myofibroblast remodeling resolved liver fibrosis

**Fig. 5:**
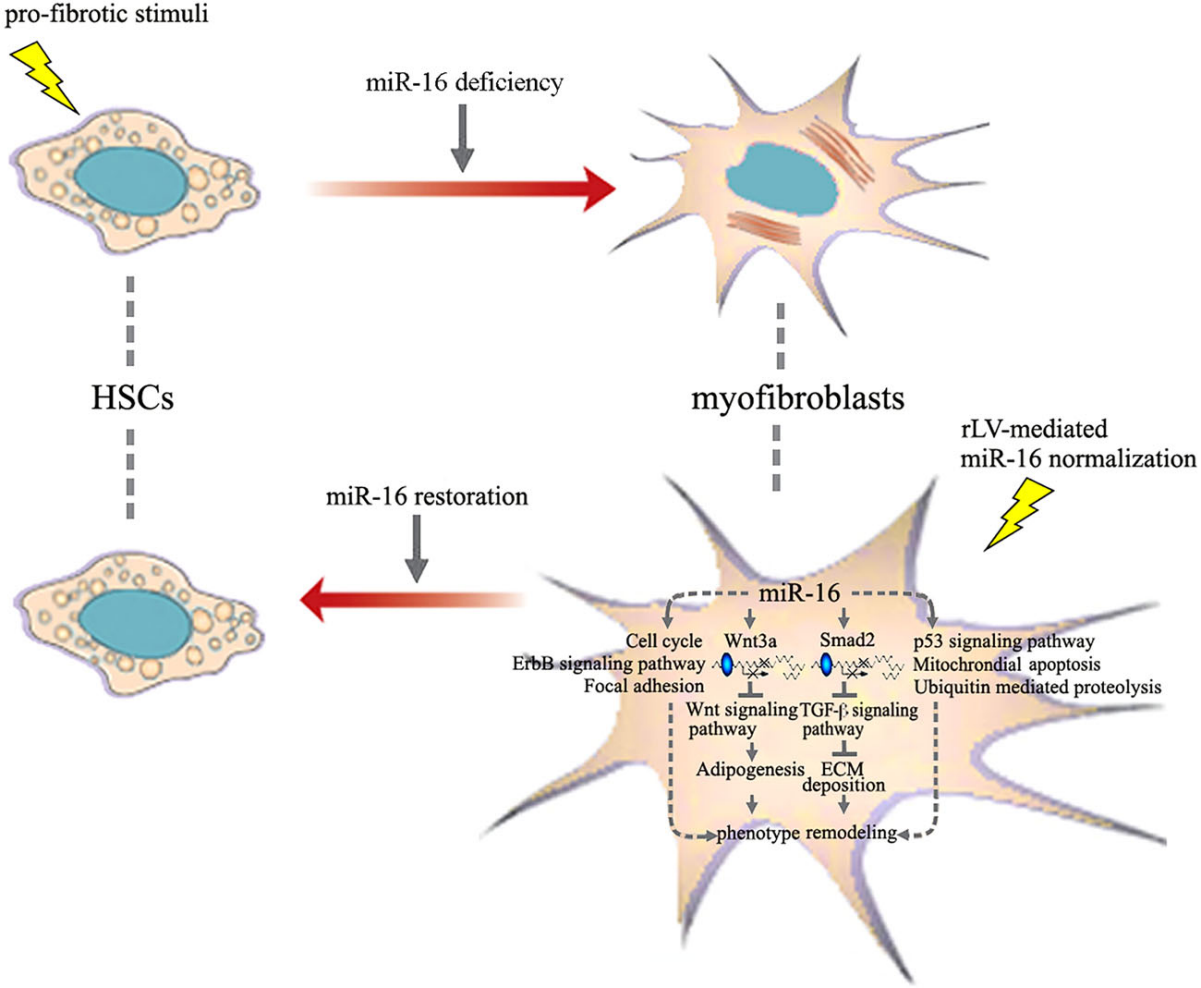
Proposed model depicting the effect of miR-16 on cell fate determination of myofibroblasts. miR-16 deficiency underlies the trandifferentiation from HSCs to myofibrobalsts after pro-fibrotic stimuli, e.g., oxidative stress. In contrast, myofibroblast-targeted miR-16 normalization orchestrates a set of signaling pathways by pleiotropic regulation of key genes. After the regulation of miR-16, both Smad2 and Wnt3a undergo mRNA degradation and translational inhibition. Integrated effect of miR-16 on signaling pathways, i.e., TGF-β, Wnt, and Mitochondrial apoptosis, abrogates characteristic functions of myofibroblasts, including collagen production and inhibition of adipogenesis, etc. Phenotype remodeling from myofibroblasts to HSC-like cells is resultantly induced.

**Fig. 6:**
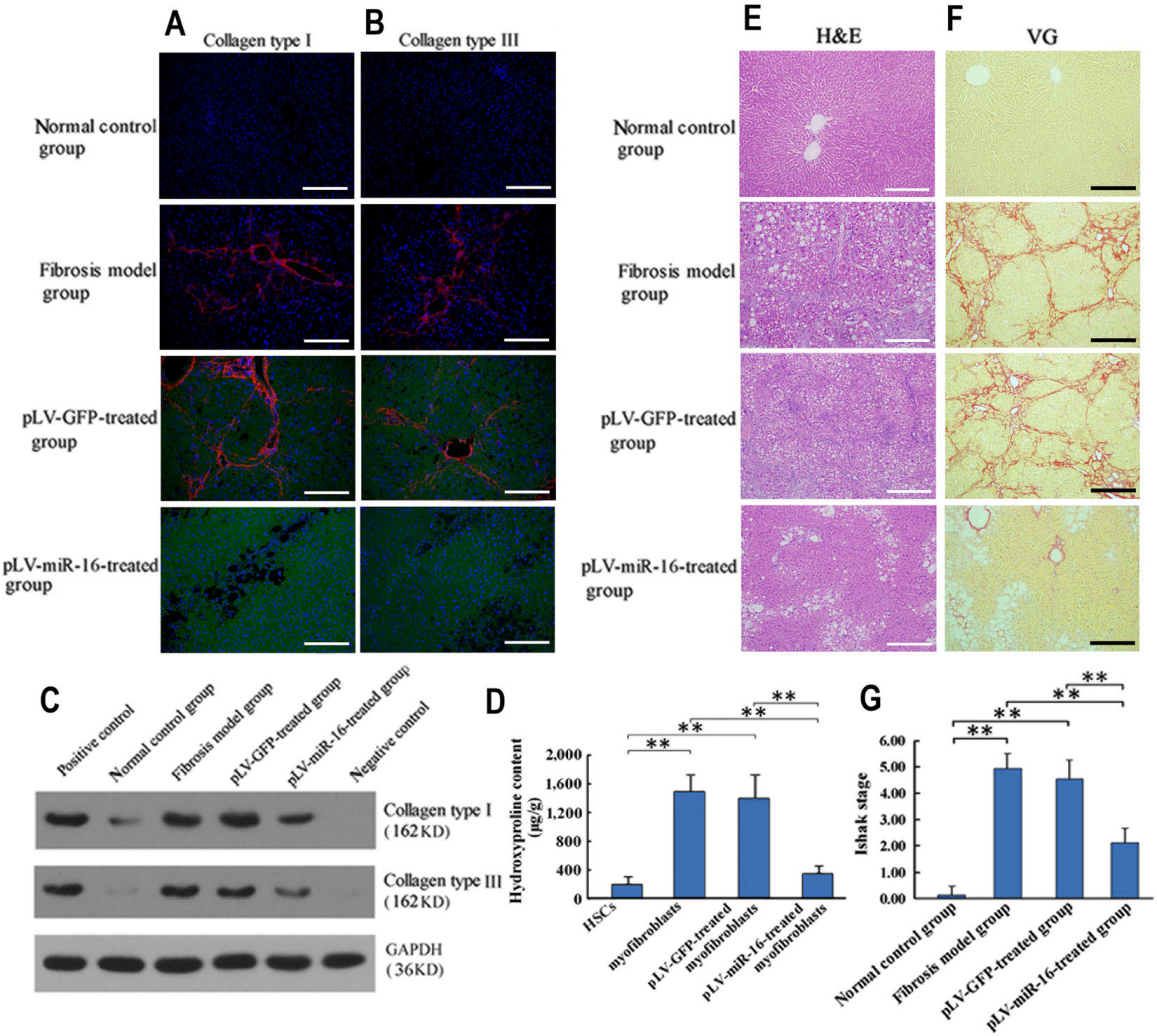
miR-16-dependent phenotype remodeling of myofibroblasts leads to the fibrosis resolution in liver. **a**–**c** Immunofluorescent staining for the deposition of collagen type I (**a**), III (**b**), and immunoblot for their levels (**c**) in the liver of normal control, fibrosis model, pLV-GFP-treated, and pLV-miR-16-treated groups, respectively (scale bar: 200 μm). Blue, red, and green signals represent the nucleus, collagen, and GFP, respectively. Representative images are shown from randomly taken pictures of liver sections in each group (nine pictures per rat, five to six rats per group). **d** Hydroxyproline content in the four groups of liver as in **a**. **e**, **f** Representative images of H&E (**e**) and VG (**f**, collagen deposition) staining (scale bar: 500 μm) in the four groups of liver as in **a**. **g** Ishak staging demonstrates the result of myofibroblasts differentiation that resolves liver fibrosis. Values are expressed as means ± SD (ANOVA) of the scoring under low-power (×100) field (nine pictures per rat, five to six rats per group). *P < 0.05, **P < 0.01.

Figure 5 shows the miR-16-mediated phenotype remodeling of myofibroblasts. This was associated with the downregulation of collagen type I and III (Fig. 6a–d and Supplementary Figs. S15 and S16) and dissolution of fibrous cords (Fig. 6e, f). Furthermore, the global impact of miR-16 lead to a decrease in the fibrosis stage (fibrosis model group vs. pLV-miR-16-treated group: 4.93 ± 0.58 vs. 2.11 ± 0.57, *P* < 0.01; pLV-GFP-treated group vs. pLV-miR-16-treated group: 4.53 ± 0.73 vs. 2.11 ± 0.57, *P* < 0.01) (Fig. 6g). However, other markers of hepatic injury (hepatosteatosis, inflammation, etc.) were not affected by the miR-16 expression.

## Discussion

miR-16 has been extensively studied as a regulator of cell phenotype^13,30,31^. Its deficiency demonstrates close association with HSC-myofibroblast transdifferentiation^13^. However, the global effect of miR-16 on cellular morphology and function remains to be understood. In this study, we observed the differential expression of multiple phenotype-determining genes upon the restoration of miR-16 expression at 48 h in myofibroblasts. HSC-specific adipogenic genes, including *RXRα*^32–34^, *PPARγ*^32–35^, *CEBPα*^32,36,37^, *CEBPθ*^2,32^, *Fabp6*^2,32^, *Fabp7*^2,32^, and *Rbp2*^38,39^, etc., were upregulated with the expression of miR-16. miR-16 also downregulated major fibrosis-inducing cytokines and related genes (*TGFβ2*^40^, *LTBP4*^41^, etc.) and collagens (*Col1a1*^42^, *Col3a1*^42^, and *Col4a5*^43^, etc.), whereas it upregulated fibrolysis-dependent matrix metallopeptidases (*MMP-2*^44^, etc.). Furthermore, the decreased expression of *CCND1*^45^, *CCNY*^46^, *Cdc20*^47^, *Cdc42*^48^, *Cdc42ep1*^49,50^, and *Cdc42ep2*^49^, together with the elevated transcription of *CDKN1A*^51^, each holding a critical place in cell cycle, reflected the inhibition of proliferation. Genes related to mitochondrial apoptosis, including *Bcl2l13*^52^, *Casp3*^53^, and *Casp9*^54^, etc., were overexpressed with increasing expression of miR-16. Attenuation of apoptosis resistance, the biological property of myofibroblasts, was suggested. miR-16 expression reversed the transcriptional profile of myofibroblasts to that of HSCs. The stable expression of miR-15b, another important member of miR-16 cluster, during 0–48 h convinced the miR-16 property of transcriptional regulation.

Signaling pathways regulated by miR-16 were acquired by the transcriptomic findings and two predicted target sets using DAVID-based data mapping. In contrast to the limited intersection between miR-16-regulated genes and predicted target genes, different sets of signaling pathways shared a large proportion of components. Therefore, synergistic regulation of a certain set of signaling pathways underlay the transcriptomic regulation of miR-16. Functional categorization using KEGG further outlined the global action of miR-16 through the intersected set of signaling pathways. By percentage, Cancers (27.91%), Signal Transduction (11.63%), Cell Growth and Death (9.30%), immune system (6.98%), Cell Communication (4.65%), and Lipid Metabolism (4.65%), etc. were classified to be the most significant functions of miR-16-regulated signaling pathways. This is in accordance with the function of miR-16 as described in other studies^13,21,55^.

We generated a pathway–pathway interaction network to identify the central signaling pathways that mediated the global effect of miR-16. The signaling pathways most regulated included the Wnt signaling pathway, TGF-β signaling pathway, p53 signaling pathway, ErbB signaling pathway, Apoptosis, Cell cycle, Focal adhesion, and Ubiquitin-mediated proteolysis. Among these, the signaling pathways related to TGF-β^56^, Wnt^57^, Apoptosis^13^, Cell cycle^58^, p53^59^, and Focal adhesion^60^ are involved in the transdifferentiation of HSCs to myofibroblasts. Thus, these pathways were proposed as the key and cooperative mediators for miR-16’s action on myofibroblasts.

To verify the established action model for miR-16, the key signaling pathways and predicted target genes were integrated and functionally analyzed. SMAD2 was a direct target of miR-16. There was a decrease in the mRNA and protein levels of SMAD2 with the increase in miR-16 levels, suggesting the negative effect of miR-16 on TGF-β signaling in myofibroblasts. This resulted in the inhibited expression and secretion of collagen types I and III. Moreover, miR-16-mediated suppression of TGF-β signaling pathway resulted in cell cycle arrest and inhibition of proliferation in myofibroblasts^61^. miR-16 also regulated Wnt signaling in these cells: Wnt3a, the activator of Wnt signaling pathway and anti-adipogenic morphogen^62^, was complementarily downregulated by miR-16. Subsequently, the expression of adipogenic TFs in HSC-like cells, which were epigenetically repressed in myofibroblasts in a β-catenin-dependent manner, was restored in the absence of Wnt-induced accumulation of β-catenin. Abolishment of apoptosis resistance and active proliferation reflected the other aspects of miR-16’s role, yet miR-16-based phenotypical remodeling underlies the outcome of most part of myofibroblast population. These findings with target-knockdown-based recapitulative and miR-16-inhibitor-based antagonistic proofs provided an explanation for the simultaneous effects of miR-16 on multiple myofibroblast-dependent signaling pathways, and in turn adjusting of the whole pathway–pathway network through “crosstalk^63^”.

HSC-to-myofibroblast transdifferentiation takes place upon the cooperation of multiple signaling pathways. Signaling pathways of TGF-β, Phosphatidylinositol-3-kinase/Akt, mitogen-activated protein kinase (MAPK)/extracellular signal-regulated kinase (ERK), etc. contribute to the imbalance of ECM synthesis/degradation^2,55,64–66^. In addition, signaling pathways of Cell cycle, TGF-β, MAPK/ERK, etc. enable the over-proliferation of myofibroblasts^2,58,61,67^. Abnormal apoptosis is attributed to signaling pathways of Wnt, p53, and Apoptosis, etc.^2,13,68,69^ The chemotactic and cell–ECM interaction properties correlated with signaling pathways of Toll-like receptor and Focal adhesion^2,60,70^. This redundant and interactive network of signaling pathways makes it difficult to reverse the phenotype of myofibroblasts by traditional one-signaling pathway interference. miR-16 restoration, however, abolished the fibrosis-inducing characteristics, and reactivated the adipogenic property by signaling pathways of TGF-β and Wnt, respectively, in an integrated way. Hence, a dramatic transition from myofibroblasts to HSC-like phenotype was finally obtained, resulting in the substantial resolution of liver fibrosis.

On the other side, abrogation of miR-16 expression in HSCs by means of gene knockout or knockdown could be an alternative for revealing its pathophysiological effects. But primary HSCs experience spontaneous transdifferentiation (activation) toward myofibroblasts, with initial and permanent activation in succession, after their separation from the sinusoidal niche of liver. A progressive descending of miR-16 level occurs during the HSC-to-myofibroblast transdifferentiation^13,71,72^. Moreover, HSCs within this period demonstrate injury susceptibility to genetic engineering and lipofectamine toxicity^73,74^. Given the limitations that prevent HSCs from miR-16 knockout or knockdown, miR-16 inhibitor was employed in the present experiments so as to mimic, to some extent, the miRNA abrogation. In contrast to the regaining of HSC-like phenotype by miR-16 restoration, treatment of miR-16 inhibitor retained the myofibroblast phenotype with reactivation of miR-16’s targets and related signaling pathways.

In conclusion, miR-16 functions by synergistically targeting a set of signaling pathways essential for myofibroblasts, such as Wnt and TGF-β, thereby globally altering the transcriptome, reversing fibrosis-related phenotypes, and inducing the resolution of liver fibrosis. Therefore, miR-16 is involved in cell fate determination in myofibroblast and may be a novel therapeutic target for hepatic fibrosis.

## Supplementary information

Supplementary Materials

Supplementary Figure 1

Supplementary Figure 2

Supplementary Figure 3

Supplementary Figure 4

Supplementary Figure 5

Supplementary Figure 6

Supplementary Figure 7

Supplementary Figure 8

Supplementary Figure 9

Supplementary Figure 10

Supplementary Figure 11

Supplementary Figure 12

Supplementary Figure 13

Supplementary Figure 14

Supplementary Figure 15

Supplementary Figure 16

Supplementary Table 1

Supplementary Table 2

Supplementary Table 3
