# Supplementary material for: miR-16 integrates signal pathways in myofibroblasts: determinant of cell fate necessary for fibrosis resolution
Source: Cell Death Dis. 2020 Aug 7;11(8):639. doi: 10.1038/s41419-020-02832-z (PMC7429878; doi:10.1038/s41419-020-02832-z)
Supplement: Supplementary file 1 — Supplementary Materials [file 41419_2020_2832_MOESM1_ESM.doc]

**Supplementary Materials**

**Materials and Methods**

**Induction of liver fibrosis**

Specific pathogens free male Sprague-Dawley rats (6-8w, 180g-240g) licensed by Experimental Animal Center of Chinese Academy of Science (Shanghai, China) were employed in the experiments. After acclimation for a week, they were divided into 2 groups at random, including control group (n=6) and fibrosis model group (n=20). Random Number Generators in SPSS was used to randomly group the experimental animals. No blinding was employed because of the toxic manifestations caused by carbon tetrachloride (CCl_4_) exposure. All rats were housed in the specific pathogen free animal unit, and permitted *ad libitum* consumption of water and diet.

Besides the double dose in the first injection, rats of the fibrosis model group were subcutaneously injected with 40% CCl_4_(0.3ml/kg) in olive oil twice weekly for 8 weeks^1^. Those in the normal group were treated with the same volume of olive oil in the same way. Hepatic histopathology was assessed on the basis of H&E and VG stained sections by 2 pathologists who were not aware of the whole experiment. The study had been approved by the Ethics Committee of Xinhua Hospital and conducted in compliance with ethical regulations of Helsinki Declaration.

**Isolation and identification of hepatic stellate cells (HSCs) and myofibroblasts**

Specific pathogens free adult male Sprague-Dawley rats (12-16w, 400-500g, Experimental Animal Center of Chinese Academy of Science, China) were employed in the experiment. After anesthesia*, in situ* serial infusion was performed *via* the portal vein with D-Hank’s solution and perfusion medium (Hank’s medium containing 0.5 g/L collagenase IV and 1 g/L pronase E). Then the liver was broken into pieces and re-digested with collagenase IV and DNase. Finally, HSCs were separated from the cell suspension by single-step density gradient centrifugation with 180 g/L Nycodenz (Sigma-Aldrich, St. Louis, MO, U.S.A.)^2^.

Adult male Sprague-Dawley rats with experimental liver fibrosis were also chosen for the isolation of myofibroblasts. Livers were separated from CCl_4_-treated rats, and then subjected to mince and type IV collagenase/Pronase E/DNAse digestion. Thereafter, myofibroblasts were purified by sequential centrifugation through 13% (v/v) and 11% (v/v) Nycodenz^3^.

After informed consent and institutional approval following the Declaration of Helsinki, human myofibroblasts were obtained from 3 patients with cirrhosis undergoing liver transplantation. While HSCs were obtained from normal tumor-adjacent liver tissues of 3 patients undergoing cholangiocarcinoma resection. In brief, 10 to 20 g of liver tissue was sliced and digested by 0.4% (W/V) Collagenase P in DMEM for 1-2 hours. The dissociated HSCs or myofibroblasts were gathered from cell suspension by density gradient centrifugation with 11% (v/v) Nycodenz, 13% (v/v) and 11% (v/v) Nycodenz, respectively^4^.

Freshly isolated HSCs and myofibroblasts were seeded for homogeneity over 24 hours. RT-QPCR for albumin (ALB), CD31, CD68, and cytokeratin-19 (CK-19) was performed to exclude the contamination of hepatocytes, cholangiocytes, Kupffer cells and endothelial cells, respectively (Table S1). The percentage of living HSCs and myofibroblasts was over 95% as defined by trypan blue staining (Sigma-Aldrich, St. Louis, MO, U.S.A.). Their purity was up to 90% when assessed by light microscopic appearance and characteristic autofluorescence, which reflected the droplet containing vitamin A in cytoplasm. The phenotype of HSCs and myofibroblasts was evaluated by Oil red O staining, together with the expression of biomarkers (α-SMA, desmin)^5^.

In result, HSCs, which were separated from normal liver, manifested their quiescent phenotype with a small spherical appearance (Fig. S1, S2A), characteristic blue-green autofluorescence from vitamin A-rich oil droplets (Fig. S2B), and oil red O staining of lipid droplets (Fig. S2C). In contrast, myofibroblasts isolated from fibrotic liver, which was induced by CCl_4_ administration for 8 weeks (Fig. S1), presented an active growth pattern and spindle-like shape with α-SMA expression (Fig. S2). The mycoplasma contamination of HSCs and myofibroblasts had been tested and excluded using the Mycoplasma Detection Kit (R&D Systems, Inc. Minneapolis, MN, U.S.A.) according to the manufacturer’s instructions.

**Lentivirus**

Precursor rno-miR-16 (pre-rno-miR-16) (Gene Accession no MI0000844, NCBI Reference Sequence: NR_031817.1) was amplified from genome of PC12 cells according to miRBase database. The primer sequences were: 5’-ATAGAATTCTTGTATTATTATGTTTGGAC-3’ (sense), 5’-ATAGGATCCAAATTATACTAGCAGGA-3’ (antisense). Packaging and production of lentivirus were performed according to the manufacturer’s protocol. Detailedly, double-stranded oligonucleotides encoding pre-miR-16 or the control sequence (pre-miR-16 with ’GGGGGG’-replaced seed sequence) were annealed and inserted into the pCDH-CMV-MCS-EF1-copGFP miRNA expression vector (System Biosciences, Mountain View, CA, U.S.A.), respectively. The new miRNA expression vectors (pCDH-CMV-MCS-EF1-copGFP-miR-16, pCDH-CMV-MCS-EF1-copGFP) and Lentivirus Package plasmid mix (System Biosciences, SBI) were co-transfected into 293TN cells with Lipofectamine 2000 (Invitrogen, Carlsbad, CA, U.S.A.)^6^ (Fig. S3). The culture supernatants were collected, concentrated, and used as a virus stock. The viral titer was determined by counting GFP-positive cells after transfection.

Similarly, LV-miR-16 specific for the α-SMA-positive cells was constructed by pCDH-CMV-MCS-EF1-copGFP miRNA expression vector, Lentivirus Package plasmid mix and sequence of α-SMA promoter.

**Microarray hybridization**

Total RNA was extracted and purified using RNeasy mini kit (QIAGEN, GmBH, Duesseldorf, Germany), and then checked for a RIN number to inspect RNA integration by Agilent Bioanalyzer 2100 (Agilent technologies, Santa Clara, CA, U.S.A.). Afterwards, total RNA was amplified, labeled and purified by GeneChip 3’IVT Express Kit (Affymetrix, Santa Clara, CA, U.S.A.) to obtain biotin labeled cRNA. Array hybridization and wash were performed using GeneChip® Hybridization, Wash and Stain Kit (Affymetrix, Santa Clara, CA, U.S.A.) in Hybridization Oven 645 (Affymetrix, Santa Clara, CA, U.S.A.) and Fluidics Station 450 (Affymetrix, Santa Clara, CA, U.S.A.). Slides were finally scanned by GeneChip® Scanner 3000 (Affymetrix, Santa Clara, CA, U.S.A.) and Command Console Software (Affymetrix, Santa Clara, CA, U.S.A.) with default settings. The values were log2 transformed.

All the MIAME-compliant microarray data had been deposited in the Gene Expression Omnibus (GEO) database (GSE40381). Six transcripts among all differentially expressed ones, *Cav1*, *ICAM1*, *Mmp2*, *Ezr*, *Casp3* and *Col1a1* were randomly selected for further verification by RT-QPCR^7-12^ (Table S1). RT-QPCR yielded results closely matching the microarray data for selected genes (Fig. S5).

**Bioinformatics**

(1) The alternations of transcriptome between HSCs, myofibroblasts, pLV-miR-16-treated myofibroblasts, and pLV-GFP-treated myofibroblasts were assessed by random variance model (RVM)^13^, and then subjected to unsupervised hierarchical clustering and TreeView analysis. (2) According to the prediction algorithm of miRBase ([http://microrna.sanger.ac.uk/sequences](http://microrna.sanger.ac.uk/sequences/)) or TargetScan 5.1 ([http://www.targetscan.org](http://www.targetscan.org/)) database^14,15^, target genes of miR-16 were pooled, respectively, to construct 2 sets of predicted-targets (miRanda-predicted targets, TargetScan-predicted targets). Additionally, genes statistically regulated by miR-16 were pooled and defined as the set of transcriptome-filtered targets. Intersection of these 3 gene-sets was then performed. (3) Percentage of signaling pathway members within the sets of transcriptome-filtered targets, miRanda-predicted targets and TargetScan-predicted targets was evaluated on the basis of Kyoto Encyclopedia of Genes and Genomes (KEGG) database (http://www.genome.jp/kegg)^16^. (4) Three gene-sets, namely 2 sets of predicted-targets and 1 set of transcriptome-filtered targets, were supplemented with proved targets^17-20^ and mapped to DAVID database^21^, respectively. As a result, 3 sets of corresponding signaling pathways, being miRanda-predicted pathways, TargetScan-predicted pathways and transcriptome-filtered pathways, were generated. Moreover, they were also subjected to intersection. (5) Equal probability sampling method evaluated the probability that intersection of signaling-pathway sets was obtained by random sampling^22^. (6) Signaling pathways virtually relevant to miR-16 were filtered from the intersection set by enrichment against the background^23^. (7) The roles of miR-16-regulated signaling pathways were measured by biological process-based categorization on the basis of KEGG database^16^. (8) Establishing by the annotations of KEGG database and directed graph theory topology, pathway-pathway interaction network illustrated the interaction of miR-16-regulated signaling pathways^24^. (9) Twenty percent of signaling pathways, which were regulated by the most number of signaling pathways (>3) within the pathway-pathway interaction network, were classified as the critical nodes of network. (10) High enrichment GOs were mined from miR-16-regulated genes in the method of Gene Ontology ([http://www.geneontology.org](http://www.geneontology.org/))^25^.

**Luciferase assay**

The 3’ untranslated region (3’UTR) segments of *SMAD2* (1875-1941bp) and *Wnt3a* (2474-2534bp), containing the putative target site for miR-16, were amplified by PCR. They were inserted, respectively, into the pMIR reporter vector (Promega, Madison, WI, U.S.A.) downstream from the stop codon of luciferase. Mutant versions of these 3’UTR segments, with mismatch to the seed sequence of miR-16, were also generated by the QuikChange II Site-Directed Mutagenesis Kit (Stratagene, La Jolla, CA, U.S.A.). miRNA Expression Reporter Vectors, with the wild versions (pMIR-SMAD2-3’UTR, pMIR-Wnt3a-3’UTR) and mutant versions of *Wnt3a*, *SMAD2* 3’UTR (MIR-SMAD2-mutant3’UTR, MIR- Wnt3a -mutant3’UTR), were then constructed.

For luciferase reporter assays, 293 cells were transiently transfected with wild-type or mutant pMIR reporter vector, pGL3-control vector (Promega, Madison, WI, U.S.A.) and miR-16. Twenty-four hours after transfection, firefly and renilla luciferase activities were measured consecutively by dual-luciferase Reporter Assay System (Promega, Madison, WI, U.S.A.) using LB9507 Single Tube Luminometer (Berthold Technologies, Bad Wildbad, Germany)^26^.

**RT-QPCR**

mRNA was subjected to RT reaction by ExScript RT reagent kit (TAKARA, Tokyo, Japan) and Real-time PCR by [Premix Ex Taq](http://www.takara.com.cn/?action=Page&Plat=pdetail&newsid=842&subclass=1) (TAKARA, Tokyo, Japan) using Applied Biosystems 7500 Fast Real-Time PCR System (Applied Biosystems, Foster City, CA, U.S.A.) according to the manufacture’s protocol. Primers for *miR-16* *^5^, Acta* *^5^, ALB ^5^, Casp3* ^11^*, Cav1* ^7^*, CD31* *^5^, CD68* *^5^, CEBPA* ^27^*, CK19* *^5^, Col1a1* ^12^*, Col3a1* ^12^*, CTNNB* ^28^*, Des* *^5^, Ezr* ^10^, *ICAM1* ^8^, *Mmp2* ^9^, *PPARG* ^29^*, Rxra* ^30^*, SMAD2,* and *Wnt3a* ^31^ were described previously.

**Western blot**

Total proteins of cells and liver tissue was exacted by Mammalian Protein Extraction Reagent (Pierce, Rockford, IL, U.S.A.) and determined by Pierce BCA Protein Assay Kit (Pierce, Rockford, IL, U.S.A.). Protein samples were then loaded on SDS-PAGE gel, separated electrophoretically and transferred to polyvinilidine difluoride membranes (Millipore, Watford, Hertfordshire, U.K.). After the non-fat dry milk (NFDM) blocking, primary antibodies of anti-Smad2 (anti-rat Smad2 (3103) 1: 1000, Cell Signaling Technology, Danvers, MA, U.S.A.; anti-human Smad2 (sc-101153) 1: 400, Santa Cruz Biotechnology, Santa Cruz, CA, U.S.A.), anti-Wnt3a (anti-rat Wnt3a (ab19925) 1: 1000, Abcam, Cambridge, MA, U.S.A.; anti-human Wnt3a (ab19925) 1: 600, Abcam, Cambridge, MA, U.S.A.), anti-β-catenin (anti-rat β-catenin (AF1329) 1: 1000, R&D Systems, Minneapolis, MN, U.S.A.), anti-C/EBPα (anti-rat C/EBPα (2843) 1: 1000, Cell Signaling Technology, Danvers, MA, U.S.A.), anti-PPARγ (anti-rat PPARγ (ab27649) 1: 300, Abcam, Cambridge, MA, U.S.A.), anti-RXRα (anti-rat RXRα (5388) 1: 800, Cell Signaling Technology, Danvers, MA, U.S.A.), anti-Col I (anti-rat Col I (C2456), 1: 2, 000, Sigma-Aldrich, St. Louis, MO, U.S.A.), anti-Col III (anti-rat Col III (C7805), 1: 1, 500, Sigma-Aldrich, St. Louis, MO, U.S.A.), anti-GAPDH (anti-GAPDH (2118), 1: 1000, Cell Signaling Technology, Danvers, MA, U.S.A.) and HRP-labeled secondary antibodies were incubated with membrane in succession. Finally, ECL Chemiluminescent Substrate Reagent Kit（Pierce, Rockford, IL, U.S.A.）was employed to record the signal on Biomax ML film (Eastman Kodak, Rochester, NY, U.S.A.). Cell Lysates of Jurkat (Clone E6-1), L Wnt-3A (ATCC® CRL-2647™), RG-C6, 3T3-L1, U937, MCF7 (ATCC® HTB-22™), and 3T3 were used as positive controls for Smad2, Wnt3a, β-catenin, PPARγ, C/EBPα, RXRα, Col I, and Col III, respectively. Negative controls were measured as well.

**Immunofluorescence**

Liver sections were fixed with 4% paraformaldehyde and permeabilized with 0.1% Triton X-100. Following nonspecific antigen blocking by 1% bovine serum albumin (BSA), they were incubated with primary antibodies (anti-rat Smad2 (sc-6200, 1: 150, Santa Cruz Biotechnology, Santa Cruz, CA, U.S.A.), anti-rat Wnt3a (sc-7382, 1: 100, Santa Cruz Biotechnology, Santa Cruz, CA, U.S.A.), anti-rat α-SMA (sc-53015, 1: 200, Santa Cruz Biotechnology, Santa Cruz, CA, U.S.A.), anti-rat Col I (sc-59772, 1: 200, Santa Cruz Biotechnology, Santa Cruz, CA, U.S.A.) and anti-rat Col III (sc-271249, 1: 200, Santa Cruz Biotechnology, Santa Cruz, CA, U.S.A.)) overnight at 4℃, and then reacted with TRITC-labeled or FITC-labeled secondary antibodies for 45 min at room temperature.

**Immunohistochemistry and immunocytochemistry**

Liver slides of 4 groups were prepared and, successively, treated by 3% H_2_O_2_, 0.01% trypsin, bovine serum albumin (BSA), anti-rat proliferating cell nuclear antigen (PCNA) (ab19166, 1: 200, Abcam, Cambridge, MA, U.S.A.) overnight at 4^∘^C, and biotin-conjugated secondary antibody (1:200; Jackson ImmunoResearch Laboratories, Inc., West Grove, PA, U.S.A.) for 30 minutes. Signals were finally visualized by DAB Substrate Kit^32^. The number of positive HSCs and myofibroblasts was counted under high-magnification field.

Similarly, HSCs and myofibroblasts were subjected to the following procedures: (1) fixation by cold acetone at -20℃, (2) blocking nonspecific antigen by BSA, (3) incubation with primary antibodies of anti-rat α-SMA (sc-53015, 1: 200, Santa Cruz Biotechnology, Santa Cruz, CA, U.S.A.) overnight at 4℃, (4) reaction with biotin-labeled secondary antibodies (1:200; Jackson ImmunoResearch Laboratories, Inc., West Grove, PA, U.S.A.), (5) visualization by DAB Substrate Kit^32^.

**Proliferation assay**

Seven groups of cells, including HSC, myofibroblast, pLV-GFP-treated myofibroblast, pLV-miR-16-treated myofibroblast, miR-16 inhibitor-treated myofibroblast, Smad2 siRNA-treated myofibroblast, and Wnt3a siRNA-treated myofibroblast, were seeded in 96 well plates at 1×10^4^ cells per well. After serum-starved incubation and then 24 h culture, the following steps were taken. (1) 10μl reagent of Cell Counting Kit-8 (CCK-8) (Dojindo, Kumamoto, Japan) was added to each well and incubated at 37℃ for 1 h. (2) The optical density (OD) was analyzed on ELx800 Microplate reader (BioTek, Winooski, VT, U.S.A.) at a test wavelength of 450 nm and a reference wavelength of 620 nm^33^.

**Flow cytometry**

The cell cycle of 7 groups were analyzed using FACS Calibur flow cytometer (BD Biosciences, San Jose, CA, U.S.A.). Detailedly, HSC, myofibroblast, pLV-GFP-treated myofibroblast, pLV-miR-16-treated myofibroblast, miR-16 inhibitor-treated myofibroblast, Smad2 siRNA-treated myofibroblast, and Wnt3a siRNA-treated myofibroblast were successively subjected to digestion, rinse with PBS, fixation with cold ethanol at -20℃, incubation with RNase at 37℃ for 15 min, and then staining with PI for 30 min in darkness. Thereafter, the cell cycle were obtained by flow cytometry^34^.

**Terminal dexynucleotidyl transferase (TdT)-mediated dUTP nick end labeling (TUNEL)**

Apoptosis rate of HSCs and myofibroblasts was determined by the TdT-DAB *in Situ* Apoptosis Detection Kit (R&D Systems, Minneapolis, MN, U.S.A.) according to the manufacturer’s instructions. In succession, liver slides of 4 groups were subjected to deparaffinization, proteinase K digestion, treatment of 1X TdT Labeling Buffer for 5 minutes, incubation with Labeling Reaction Mix at 37 °C for 1 hour, covering with Strep-HRP Solution at 37 °C for 10 minutes, and visualization with DAB Solution^35^. The number of positive HSCs and myofibroblasts was counted under high-magnification field.

**Enzyme-linked immunosorbent assay (ELISA)**

Culture supernatant of 7 groups was collected and concentrated by YM 30 Concenterator (Millipore, Watford, Hertfordshire, U.K.). Collagen type I and III in the supernatant were investigated by ELISA kits (Cusabio, College Park, MD, U.S.A.) using DENLEY DRAGON Wellscan MK 2 (Thermo Fisher Labsystems, Helsinki, Finland). Ascent software for Multiskan (Thermo Fisher Labsystems, Helsinki, Finland) was then employed to obtain the collagen concentrations.

**Ultrastructrue**

Cells (HSCs, myofibroblast) and liver sections were successively treated with 2% glutaraldehyde, 0.1 M phosphate buffer (pH 7.4, 2 h), PBS, 2% osmium-tetraoxide (4℃, 2 h) and alcohol series, then embedded in epoxy resin. Ultrathin sections (80–100 nm) were sliced, following with contrasted with uranyl acetate and lead citrate. Finally, the apoptosis-specific ultrastructure of HSCs was visualized by transmission electron microscope CM120 (Philips, Eindhoven, Netherland).

**Lentivirus treatment**

Tool of Power And Sample Size (<http://powerandsamplesize.com/Calculators/>) was used in the present study to estimate the sample size and power of animal experiments. First, the choice of ‘compare 2 proportions: 2-sample, 2-sided equality’ was selected in the panel on a basis of algorithm described previously^36^. Then the successful rate of experimental liver fibrosis and type I error (α) were defined to be 65% and 5%, respectively^37^. In result, 80% desired power was obtained in condition of a sample size of 5 rat/group. Nevertheless, there was 83.65% desired power when the sample size reached 5 rats per group.

Accordingly, adult male Sprague-Dawley rats were randomly grouping into control group normal control group (n = 6), fibrosis model group (n = 5), pLV-miR-16-treated group (n = 5) and pLV-GFP-treated group (n = 5) using Random Number Generators in SPSS. Both LV-miR-16-treated group and LV-GFP-treated group were treated by portal vein cannulation. In detail, the superior mesentery vein (SMV) was exposed by a midline incision. A 24-gauge intravenous catheter was then inserted into the SMV *via* its ramus. After fixation, the catheter was extended into the subcutaneous space by an extradural catheter, and was fixed for lentivirus delivery after adding a heparinized cap.

After the CCl_4_ administration for 4 weeks, LV-miR-16 (LV-miR-16-treated group) or LV-GFP (LV-GFP-treated group) was delivered, respectively, *in vivo* by the portal vein injection method for another 4 weeks. Meanwhile, liver fibrosis was still induced by CCl_4_ subcutaneously.

**Pathology**

Liver tissue was fixed with 4% paraformaldehyde in phosphate-buffered saline (PH 7.4). Successive treating with graded alcohol and dimethylbenzene, the specimens were embedded in paraffin. Five μm-thick sections from each block were then prepared and subjected to hematoxylin and eosin (H&E) staining, Van Gieson (VG) staining, respectively. Fibrosis staging with Ishak staging system^38^ was also independently determined by 2 pathologists who were not aware of the experiments.

**Statistics**

The statistical tests for every figure were justified and convinced to be appropriate. By biological and technical repetition, data for statistical tests met the assumptions of normal distribution. The data variation of each group was subjected to estimation, and similar variance existed between the groups under statistical comparison.

**Image**

Figure S1A, S1B and Figure 6E, 6F were taken by microscope IX71 (Olympus, Tokyo, Japan) under the following conditions: room temperature, bright field, air-mediation, magnification 40×, 1280x1024 pixels, 24-bit color depth.

Figure S2A and Left panel of Figure S5A are taken by microscope IX71 under the following conditions: room temperature, phase-contrast microscopy, air-mediation, magnification 200×, 1280x1024 pixels, 24-bit color depth.

Figure S2B is taken by microscope IX71 under the following conditions: room temperature, immunofluorescence microscopy at 328 nm, air-mediation, magnification 100×, 1280x1024 pixels, 8-bit color depth.

Figure S2C, Figure 4I, and Figure 4K are taken by microscope IX71 under the following conditions: room temperature, phase-contrast microscopy, air-mediation, magnification 400×, 1280x1024 pixels, 24-bit color depth.

Figure S2D is taken by microscope IX71 under the following conditions: room temperature, bright field, air-mediation, magnification 400×, 1280x1024 pixels, 24-bit color depth.

Right panel of Figure S5A is taken by microscope IX71 under the following conditions: room temperature, immunofluorescence microscopy with the excitation wavelength of 490 nm and the emission wavelength of 520 nm, air-mediation, magnification 200×, 1280x1024 pixels, 8-bit color depth.

Figure 4G are taken by microscope IX71 under the following conditions: room temperature, immunofluorescence microscopy, air-mediation, magnification 200×, 1280x1024 pixels, 24-bit color depth. Each panel of these figures is detected at the excitation wavelengths of 352 nm, 490 nm, 550 nm and emission wavelengths of 461 nm, 520 nm, 573 nm, respectively. Signals of hochest 33258, FITC and TRITC are then merged using Picture Merge Genius V2.7.141 (Easytools, Inc).

Figure 6A, 6B are taken by microscope IX71 under the following conditions: room temperature, immunofluorescence microscopy, air-mediation, magnification 100×, 1280x1024 pixels, 24-bit color depth. Each panel of these figures is detected at the excitation wavelengths of 352 nm, 490 nm, 550 nm and emission wavelengths of 461 nm, 520 nm, 573 nm, respectively. Signals of hochest 33258, FITC and TRITC are then merged using Picture Merge Genius V2.7.141 (Easytools, Inc).

**References**

1 Farrell, G. C. & Zaluzny, L. Hepatic heme metabolism and cytochrome P450 in cirrhotic rat liver. *Gastroenterology* **89**, 172-179, doi:10.1016/0016-5085(85)90759-0 (1985).

2 Popov, Y. *et al.* Halofuginone induces matrix metalloproteinases in rat hepatic stellate cells via activation of p38 and NFkappaB. *J Biol Chem* **281**, 15090-15098, doi:10.1074/jbc.M600030200 (2006).

3 Montosi, G., Garuti, C., Iannone, A. & Pietrangelo, A. Spatial and temporal dynamics of hepatic stellate cell activation during oxidant-stress-induced fibrogenesis. *Am J Pathol* **152**, 1319-1326 (1998).

4 Estep, J. M. *et al.* Hepatic stellate cell and myofibroblast-like cell gene expression in the explanted cirrhotic livers of patients undergoing liver transplantation. *Dig Dis Sci* **55**, 496-504, doi:10.1007/s10620-009-0919-9 (2010).

5 Guo, C. J., Pan, Q., Li, D. G., Sun, H. & Liu, B. W. miR-15b and miR-16 are implicated in activation of the rat hepatic stellate cell: An essential role for apoptosis. *J Hepatol* **50**, 766-778, doi:10.1016/j.jhep.2008.11.025 (2009).

6 Scurr, L. L. *et al.* IGFBP7 Is Not Required for B-RAF-Induced Melanocyte Senescence. *Cell* **141**, 717-727, doi:10.1016/j.cell.2010.04.021 (2010).

7 Grayson, T. H. *et al.* Vascular microarray profiling in two models of hypertension identifies caveolin-1, Rgs2 and Rgs5 as antihypertensive targets. *BMC Genomics* **8**, 404, doi:10.1186/1471-2164-8-404 (2007).

8 Koudstaal, L. G. *et al.* Brain death induces inflammation in the donor intestine. *Transplantation* **86**, 148-154, doi:10.1097/TP.0b013e31817ba53a (2008).

9 Liu, Y. *et al.* Renal medullary microRNAs in Dahl salt-sensitive rats: miR-29b regulates several collagens and related genes. *Hypertension* **55**, 974-982, doi:10.1161/hypertensionaha.109.144428 (2010).

10 Laragione, T., Brenner, M., Li, W. & Gulko, P. S. Cia5d regulates a new fibroblast-like synoviocyte invasion-associated gene expression signature. *Arthritis Res Ther* **10**, R92, doi:10.1186/ar2476 (2008).

11 Wu, J., Gorman, A., Zhou, X., Sandra, C. & Chen, E. Involvement of caspase-3 in photoreceptor cell apoptosis induced by in vivo blue light exposure. *Invest Ophthalmol Vis Sci* **43**, 3349-3354 (2002).

12 Nagai, Y. *et al.* Aldosterone stimulates collagen gene expression and synthesis via activation of ERK1/2 in rat renal fibroblasts. *Hypertension* **46**, 1039-1045, doi:10.1161/01.Hyp.0000174593.88899.68 (2005).

13 Wright, G. W. & Simon, R. M. A random variance model for detection of differential gene expression in small microarray experiments. *Bioinformatics* **19**, 2448-2455, doi:10.1093/bioinformatics/btg345 (2003).

14 Griffiths-Jones, S., Grocock, R. J., van Dongen, S., Bateman, A. & Enright, A. J. miRBase: microRNA sequences, targets and gene nomenclature. *Nucleic Acids Res* **34**, D140-144, doi:10.1093/nar/gkj112 (2006).

15 Lewis, B. P., Burge, C. B. & Bartel, D. P. Conserved seed pairing, often flanked by adenosines, indicates that thousands of human genes are microRNA targets. *Cell* **120**, 15-20, doi:10.1016/j.cell.2004.12.035 (2005).

16 Kanehisa, M., Goto, S., Kawashima, S., Okuno, Y. & Hattori, M. The KEGG resource for deciphering the genome. *Nucleic Acids Res* **32**, D277-280, doi:10.1093/nar/gkh063 (2004).

17 Martello, G. *et al.* MicroRNA control of Nodal signalling. *Nature* **449**, 183-188, doi:10.1038/nature06100 (2007).

18 Liu, Q. *et al.* miR-16 family induces cell cycle arrest by regulating multiple cell cycle genes. *Nucleic Acids Res* **36**, 5391-5404, doi:10.1093/nar/gkn522 (2008).

19 Kaddar, T. *et al.* Two new miR-16 targets: caprin-1 and HMGA1, proteins implicated in cell proliferation. *Biol Cell* **101**, 511-524, doi:10.1042/bc20080213 (2009).

20 Bhattacharya, R. *et al.* MiR-15a and MiR-16 control Bmi-1 expression in ovarian cancer. *Cancer Res* **69**, 9090-9095, doi:10.1158/0008-5472.Can-09-2552 (2009).

21 Dennis, G., Jr. *et al.* DAVID: Database for Annotation, Visualization, and Integrated Discovery. *Genome Biol* **4**, P3 (2003).

22 Alwan, H., Viswanathan, B., Rousson, V., Paccaud, F. & Bovet, P. Association between substance use and psychosocial characteristics among adolescents of the Seychelles. *BMC Pediatr* **11**, 85, doi:10.1186/1471-2431-11-85 (2011).

23 Guo, C. J. *et al.* Changes in microRNAs associated with hepatic stellate cell activation status identify signaling pathways. *Febs j* **276**, 5163-5176, doi:10.1111/j.1742-4658.2009.07213.x (2009).

24 Yi, M., Horton, J. D., Cohen, J. C., Hobbs, H. H. & Stephens, R. M. WholePathwayScope: a comprehensive pathway-based analysis tool for high-throughput data. *BMC Bioinformatics* **7**, 30, doi:10.1186/1471-2105-7-30 (2006).

25 Ashburner, M. *et al.* Gene ontology: tool for the unification of biology. The Gene Ontology Consortium. *Nat Genet* **25**, 25-29, doi:10.1038/75556 (2000).

26 Kolachala, V. L. *et al.* Adenosine 2B receptor expression is post-transcriptionally regulated by microRNA. *J Biol Chem* **285**, 18184-18190, doi:10.1074/jbc.M109.066555 (2010).

27 Desai, M., Guang, H., Ferelli, M., Kallichanda, N. & Lane, R. H. Programmed upregulation of adipogenic transcription factors in intrauterine growth-restricted offspring. *Reprod Sci* **15**, 785-796, doi:10.1177/1933719108318597 (2008).

28 Hewitt, D. P., Mark, P. J., Dharmarajan, A. M. & Waddell, B. J. Placental expression of secreted frizzled related protein-4 in the rat and the impact of glucocorticoid-induced fetal and placental growth restriction. *Biol Reprod* **75**, 75-81, doi:10.1095/biolreprod.105.047647 (2006).

29 Fang, X., Feng, Y., Shi, Z. & Dai, J. Alterations of cytokines and MAPK signaling pathways are related to the immunotoxic effect of perfluorononanoic acid. *Toxicol Sci* **108**, 367-376, doi:10.1093/toxsci/kfp019 (2009).

30 Nasrollahzadeh, J. *et al.* The influence of feeding linoleic, gamma-linolenic and docosahexaenoic acid rich oils on rat brain tumor fatty acids composition and fatty acid binding protein 7 mRNA expression. *Lipids Health Dis* **7**, 45, doi:10.1186/1476-511x-7-45 (2008).

31 Sklepkiewicz, P. *et al.* Glycogen synthase kinase 3beta contributes to proliferation of arterial smooth muscle cells in pulmonary hypertension. *PLoS One* **6**, e18883, doi:10.1371/journal.pone.0018883 (2011).

32 Cassiman, D., Denef, C., Desmet, V. J. & Roskams, T. Human and rat hepatic stellate cells express neurotrophins and neurotrophin receptors. *Hepatology (Baltimore, Md.)* **33**, 148-158 (2001).

33 Tanikawa, R. *et al.* Interaction of galectin-9 with lipid rafts induces osteoblast proliferation through the c-Src/ERK signaling pathway. *J Bone Miner Res* **23**, 278-286, doi:10.1359/jbmr.071008 (2008).

34 Guo, C. J., Pan, Q., Jiang, B., Chen, G. Y. & Li, D. G. Effects of upregulated expression of microRNA-16 on biological properties of culture-activated hepatic stellate cells. *Apoptosis* **14**, 1331-1340, doi:10.1007/s10495-009-0401-3 (2009).

35 Kivinen, P. K., Nilsson, G., Naukkarinen, A. & Harvima, I. T. Mast cell survival and apoptosis in organ-cultured human skin. *Exp Dermatol* **12**, 53-60, doi:10.1034/j.1600-0625.2003.120107.x (2003).

36 Chow S, S. J., Wang H. *Sample Size Calculations in Clinical Research. 2nd Ed. Chapman & Hall/CRC Biostatistics Series.*, 89 (2008).

37 Liu, Y. Q. *et al.* Inhibition of PDGF, TGF-beta, and Abl signaling and reduction of liver fibrosis by the small molecule Bcr-Abl tyrosine kinase antagonist Nilotinib. *Journal of Hepatology* **55**, 612-625, doi:10.1016/j.jhep.2010.11.035 (2011).

38 Prati, G. M. *et al.* Hyporesponsiveness to PegIFNalpha2B plus ribavirin in patients with hepatitis C-related advanced fibrosis. *J Hepatol* **56**, 341-347, doi:10.1016/j.jhep.2011.05.022 (2012).

**Supplementary figure legends**

Fig. S1 Establishment of liver fibrosis. (A, B) H&E (A), VG (B) staining of the normal control and fibrosis model groups, respectively (scale bar: 500μm).

Fig. S2 Isolation and identification of HSCs from the normal control group, and myofibroblasts from the fibrosis model groups. (A, B, C, D) Morphology (A, scale bar: 50μm), spontaneous fluorescence (B, scale bar: 100μm), Oil Red O staining (C, scale bar: 25μm) and α-SMA-specific immunohistochemical staining (D, scale bar: 25 μm) of HSCs and myofibroblasts, respectively. (E) RT-QPCR for *desmin*, *α-SMA*, *ALB*, *CD31*, *CD68*, and *CK-19*. Values are expressed as means ± S.D. ** *P* <0.01.

Fig. S3 Myofibroblasts demonstrate expressive downregulation of miR-16 in comparison to hepatic stellate cells (HSCs). Values are expressed as means ± S.D. * *P* < 0.05.

Fig. S4 Schematic representation of the lentiviral vector carrying pre-rno-miR-16 and lentiviral transfection of myofibroblasts.

Fig. S5 miR-16 restoration in myofibroblasts. (A) Light and fluorescent microscopy of myofibroblasts reflects the transfection efficiency (> 90%) of lentiviral vector at 48 h (scale bar: 100μm). (B) Expression level of miR-16 in myofibroblasts. Values are expressed as means ± S.D. * *P* < 0.05.

Fig. S6 Dynamic expression of miR-16 cluster members after the miR-16 restoration in myofibroblasts. Values are expressed as means ± S.D. * *P* < 0.05.

Fig. S7 Validation of microarray data by RT-QPCR. Values are expressed as means ± S.D. * *P* < 0.05, ** *P* < 0.01.

Fig. S8 Percentage of signaling pathway members within miR-16-regulated genes and miR-16-predicted-targets. Values are expressed as means ± S.D. ** *P* < 0.01.

Fig. S9 Quantification of miR-16 targets by western blot. (A) Densitometric ratios of Smad2 to GAPDH in groups of HSC, myofibroblast, pLV-GFP-treated myofibroblast, pLV-miR-16-treated myofibroblast, miR-16 inhibitor-treated myofibroblast, and Smad2 siRNA-treated myofibroblast. (B) Densitometric ratios of Wnt3a to GAPDH in groups of HSC, myofibroblast, pLV-GFP-treated myofibroblast, pLV-miR-16-treated myofibroblast, miR-16 inhibitor-treated myofibroblast, and Wnt3a siRNA-treated myofibroblast. Values are expressed as means ± S.D. * *P* < 0.05, ** *P* < 0.01.

Fig. S10 Densitometric ratios of adipogenic transcription factors to GAPDH in HSC, myofibroblast, pLV-GFP-treated myofibroblast, pLV-miR-16-treated myofibroblast, miR-16 inhibitor-treated myofibroblast, Smad2 siRNA-treated myofibroblast, and Wnt3a siRNA-treated myofibroblast as analyzed by western blot. Values are expressed as means ± S.D. * *P* < 0.05, ** *P* < 0.01.

Fig. S11 Densitometric ratios of miR-16 targets to GAPDH in human HSCs (Normal group) and myofibroblasts (Fibrosis group) as analyzed by western blot. Values are expressed as means ± S.D. * *P* < 0.05.

Fig. S12 Densitometric ratios of miR-16 targets to GAPDH in rat HSCs (Normal control group) and myofibroblasts (Fibrosis model group, pLV-GFP-treated group and pLV-miR-16-treated group) as analyzed by western blot. Values are expressed as means ± S.D. * *P* < 0.05, ** *P* < 0.01.

Fig. S13 Schematic representation of the lentiviral vector carrying α-SMA promoter and pre-rno-miR-16, and the *in vivo* lentiviral treatment of liver fibrosis.

Fig. S14 Numerical analysis for the positive cells of Smad2 and Wnt3a, respectively, in normal control, fibrosis model, pLV-GFP-treated and pLV-miR-16-treated groups. Values are expressed as means ± S.D. ** *P* < 0.01.

Fig. S15 Densitometric ratios of collagens to GAPDH in rat HSCs (Normal control group) and myofibroblasts (Fibrosis model group, pLV-GFP-treated group and pLV-miR-16-treated group) as analyzed by western blot. Values are expressed as means ± S.D. * *P* < 0.05, ** *P* < 0.01.

Fig. S16 Areametric analysis of collagen type I and III, respectively, in normal control, fibrosis model, pLV-GFP-treated and pLV-miR-16-treated groups. Values are expressed as means ± S.D. ** *P* < 0.01.
