# Supplementary figures and images for: miR-16 integrates signal pathways in myofibroblasts: determinant of cell fate necessary for fibrosis resolution
Source: Cell Death Dis. 2020 Aug 7;11(8):639. doi: 10.1038/s41419-020-02832-z (PMC7429878; doi:10.1038/s41419-020-02832-z)

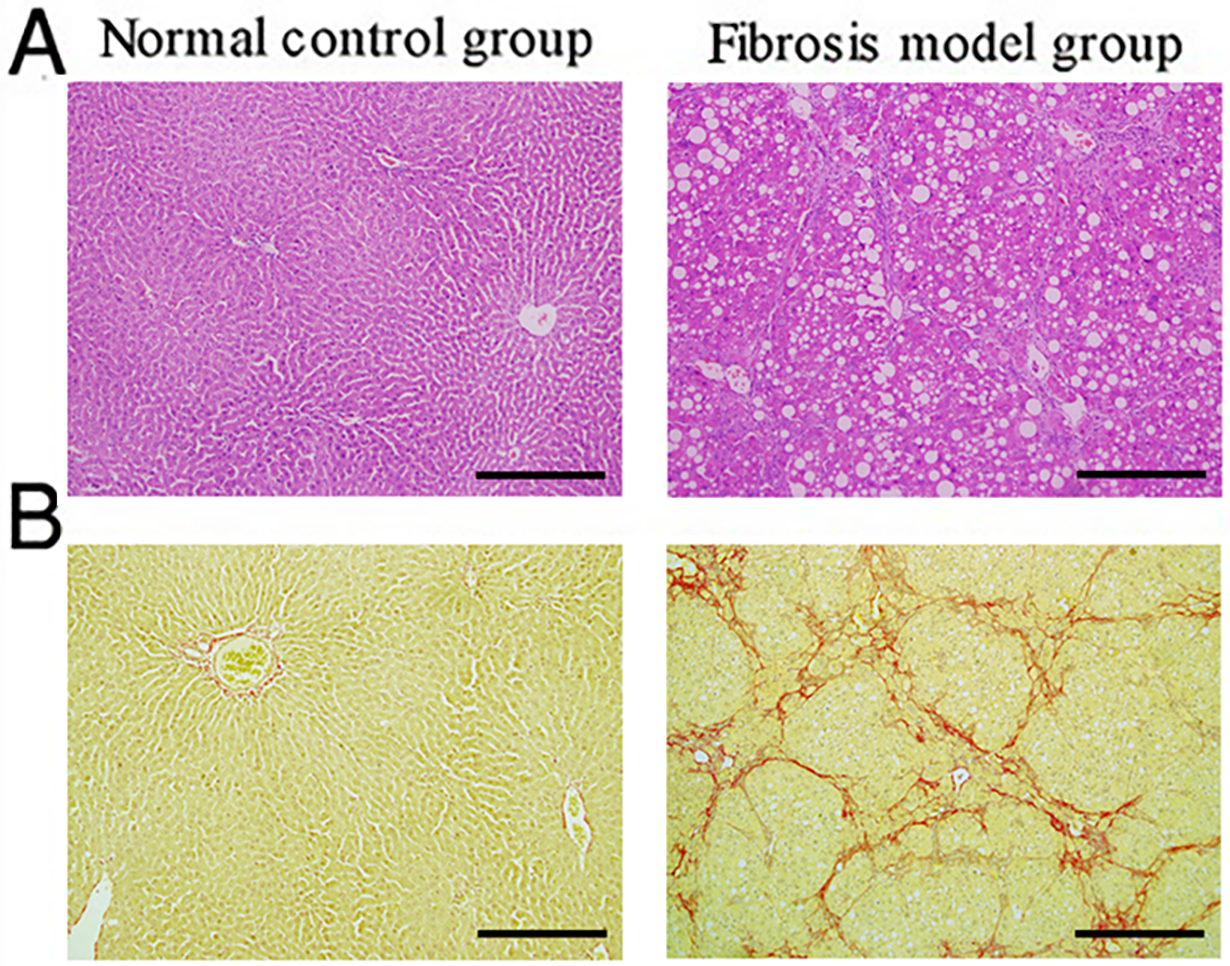

Supplement: Supplementary file 2 — Supplementary Figure 1 [file 41419_2020_2832_MOESM2_ESM.png]

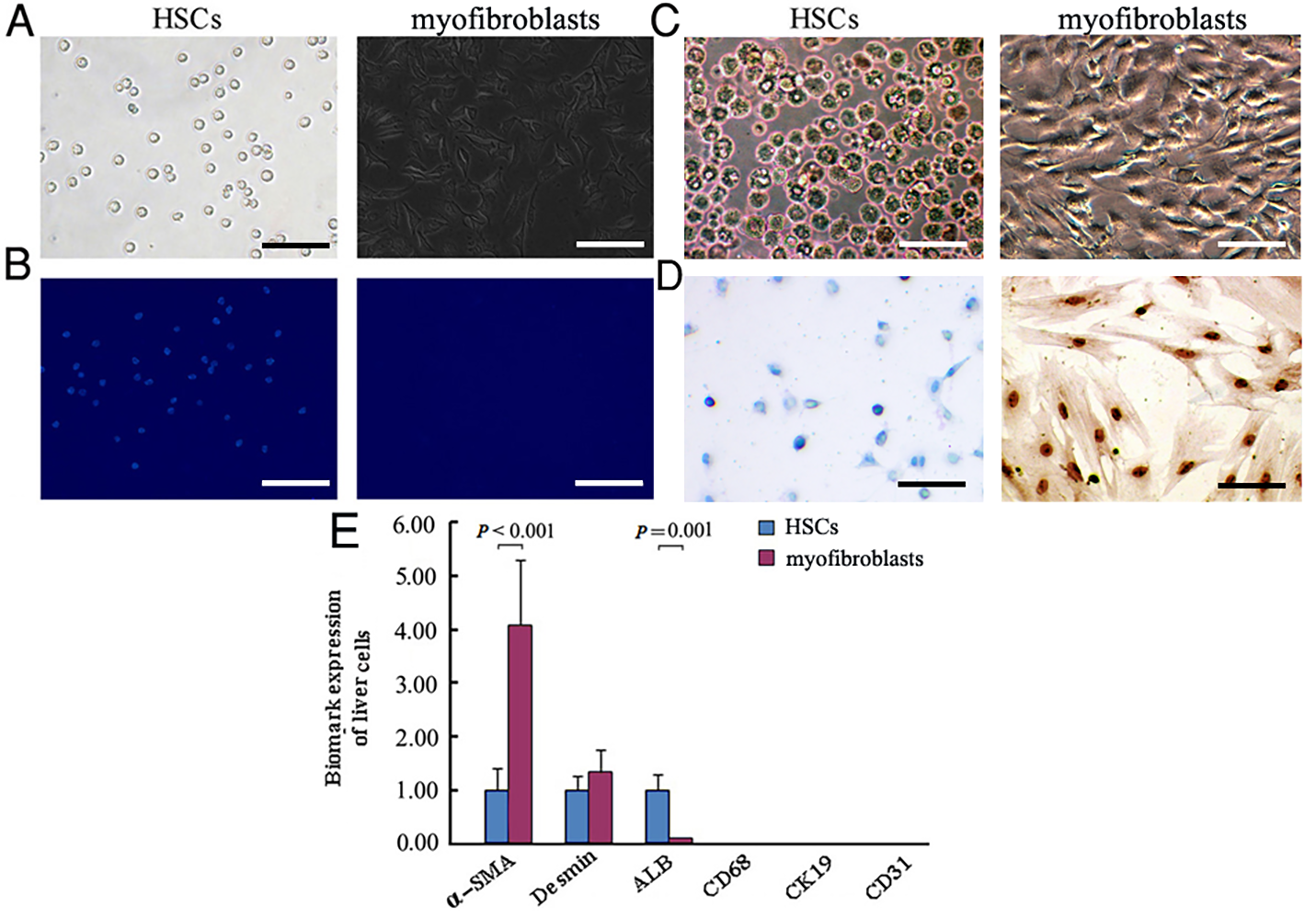

Supplement: Supplementary file 3 — Supplementary Figure 2 [file 41419_2020_2832_MOESM3_ESM.png]

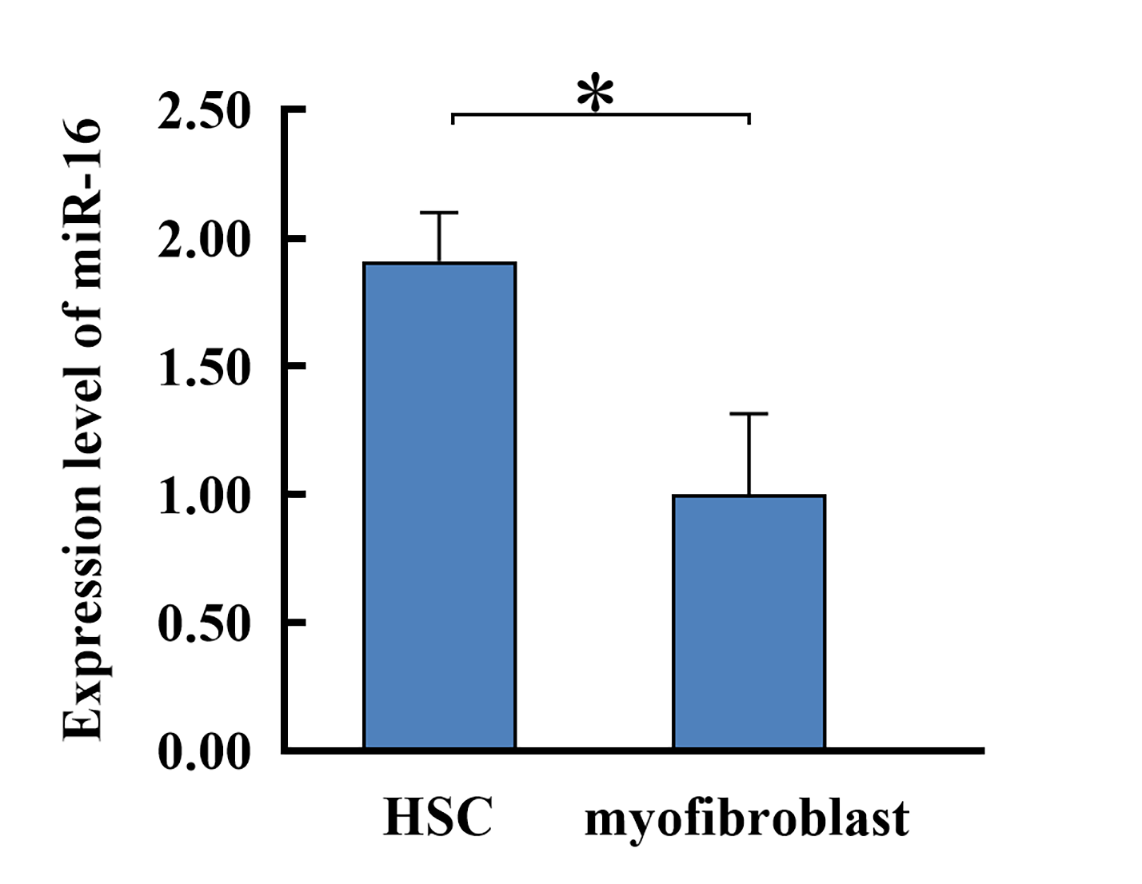

Supplement: Supplementary file 4 — Supplementary Figure 3 [file 41419_2020_2832_MOESM4_ESM.png]

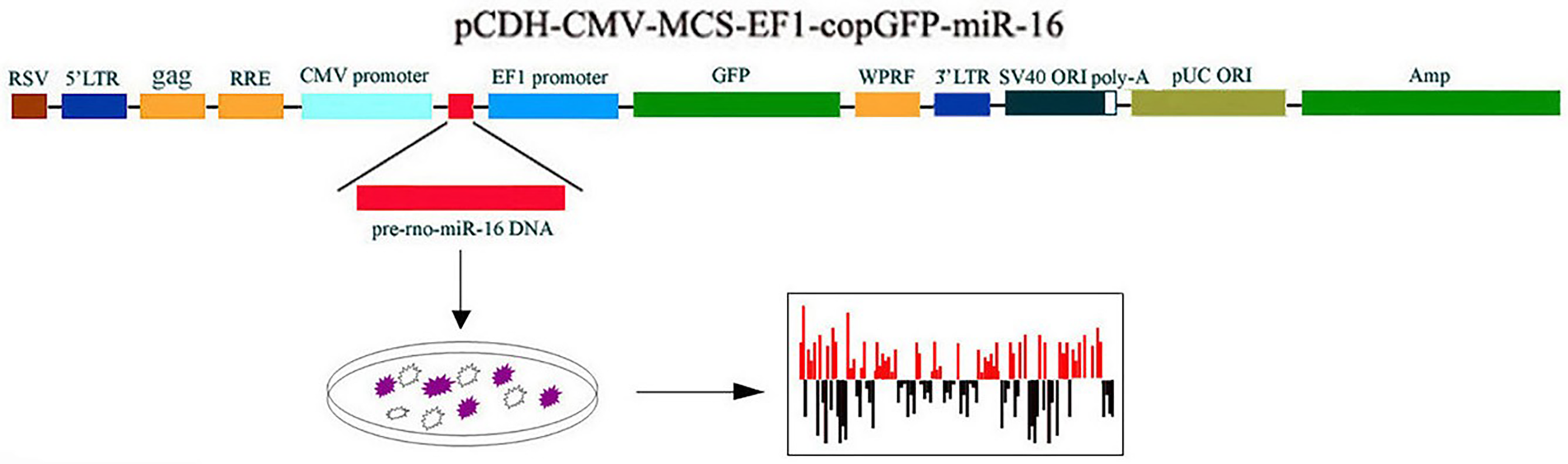

Supplement: Supplementary file 5 — Supplementary Figure 4 [file 41419_2020_2832_MOESM5_ESM.png]

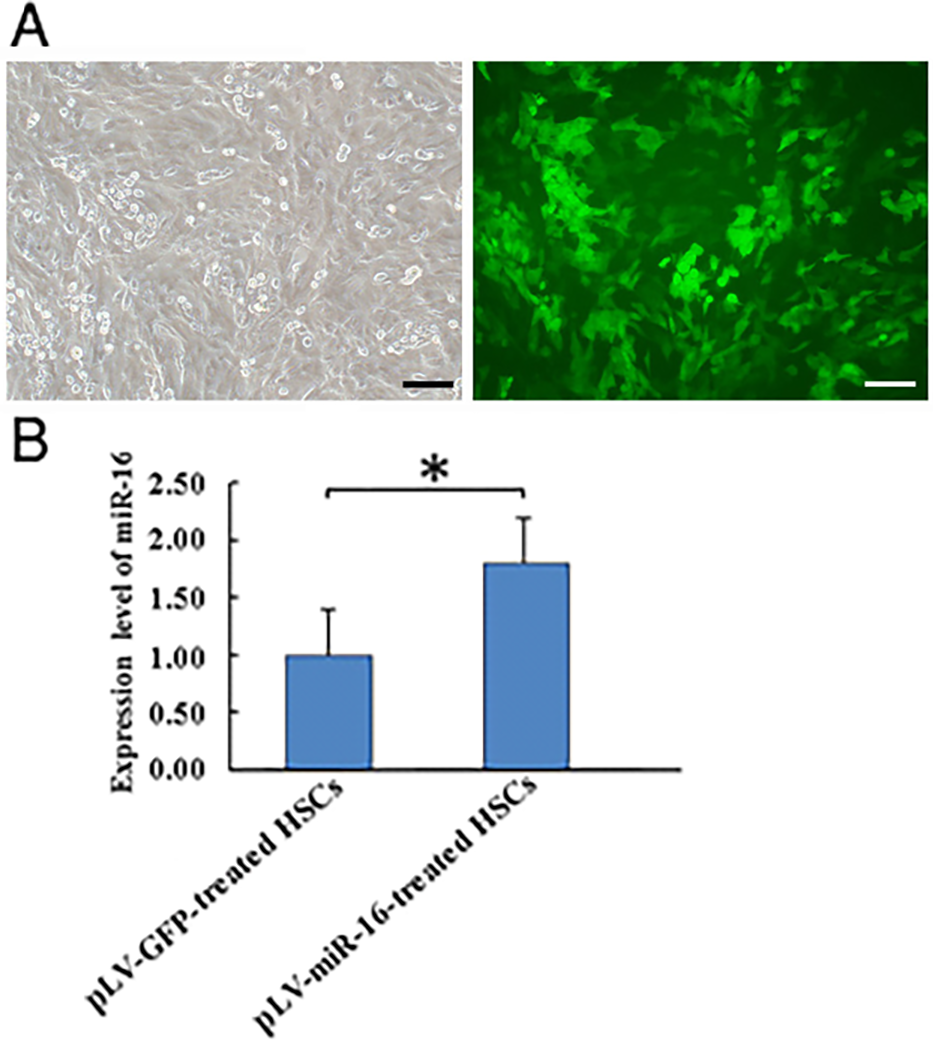

Supplement: Supplementary file 6 — Supplementary Figure 5 [file 41419_2020_2832_MOESM6_ESM.png]

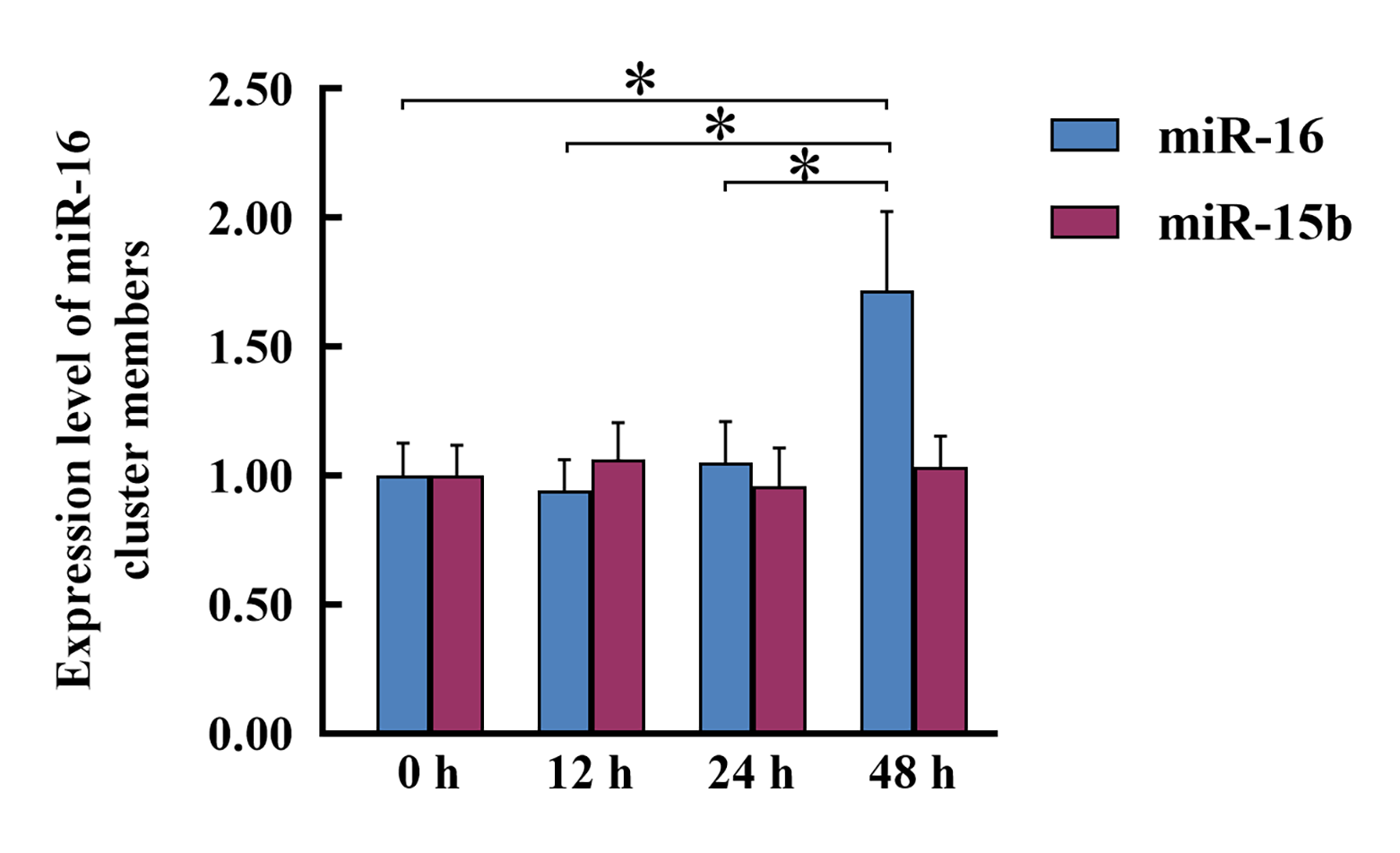

Supplement: Supplementary file 7 — Supplementary Figure 6 [file 41419_2020_2832_MOESM7_ESM.png]

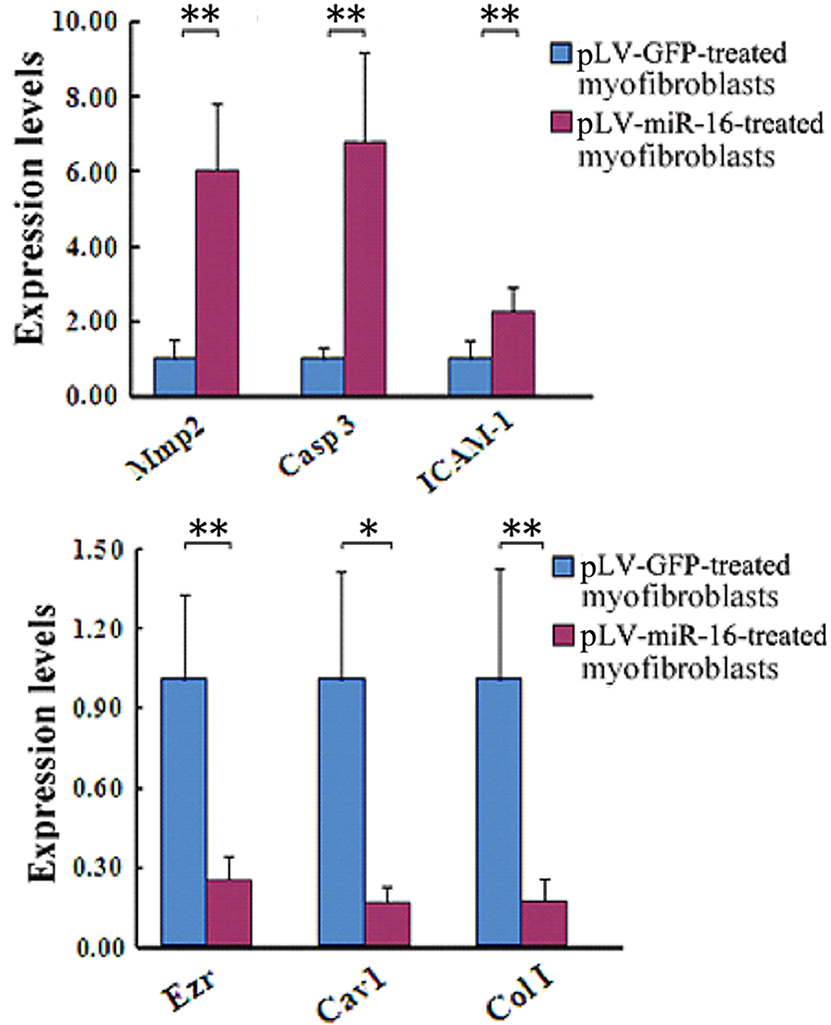

Supplement: Supplementary file 8 — Supplementary Figure 7 [file 41419_2020_2832_MOESM8_ESM.png]

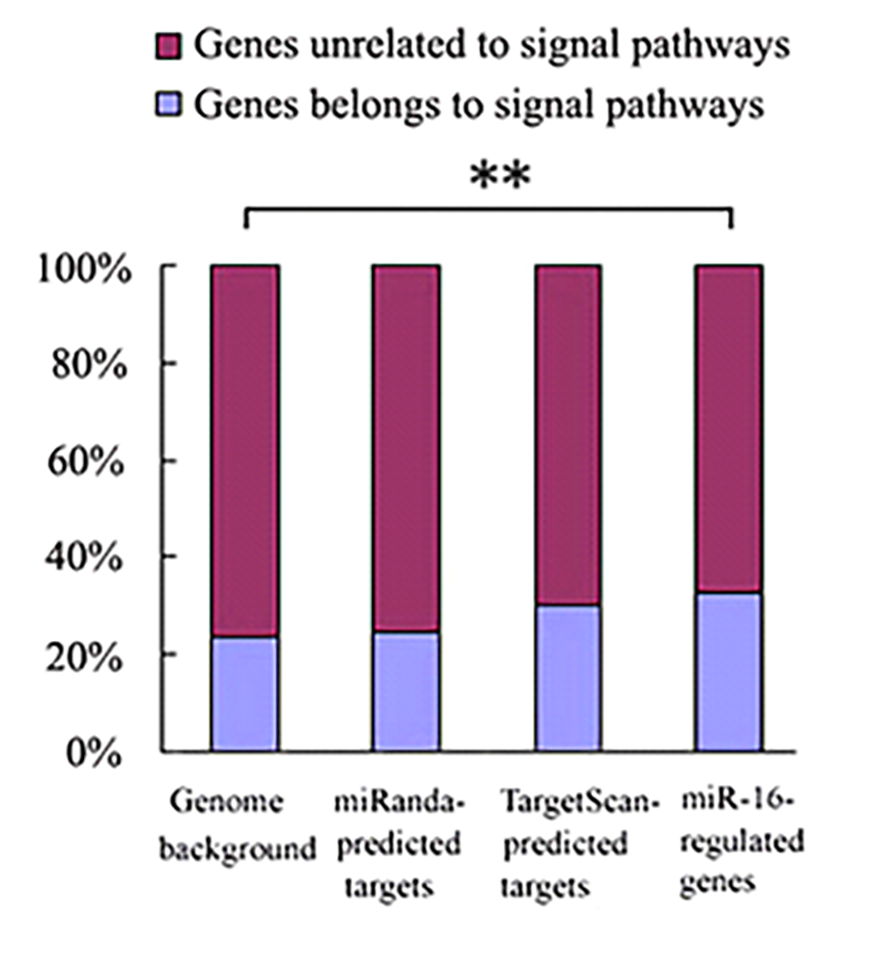

Supplement: Supplementary file 9 — Supplementary Figure 8 [file 41419_2020_2832_MOESM9_ESM.png]

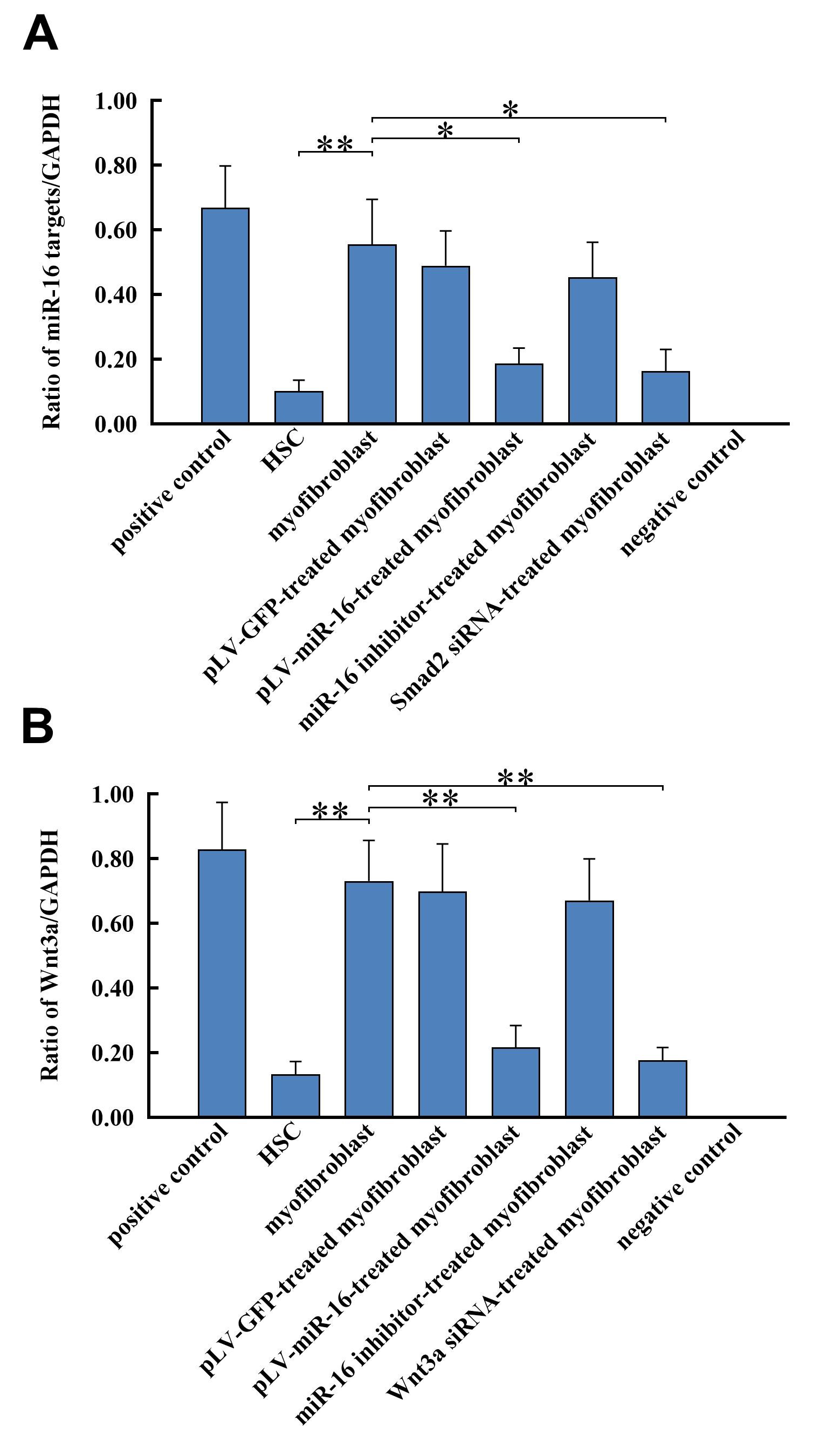

Supplement: Supplementary file 10 — Supplementary Figure 9 [file 41419_2020_2832_MOESM10_ESM.png]

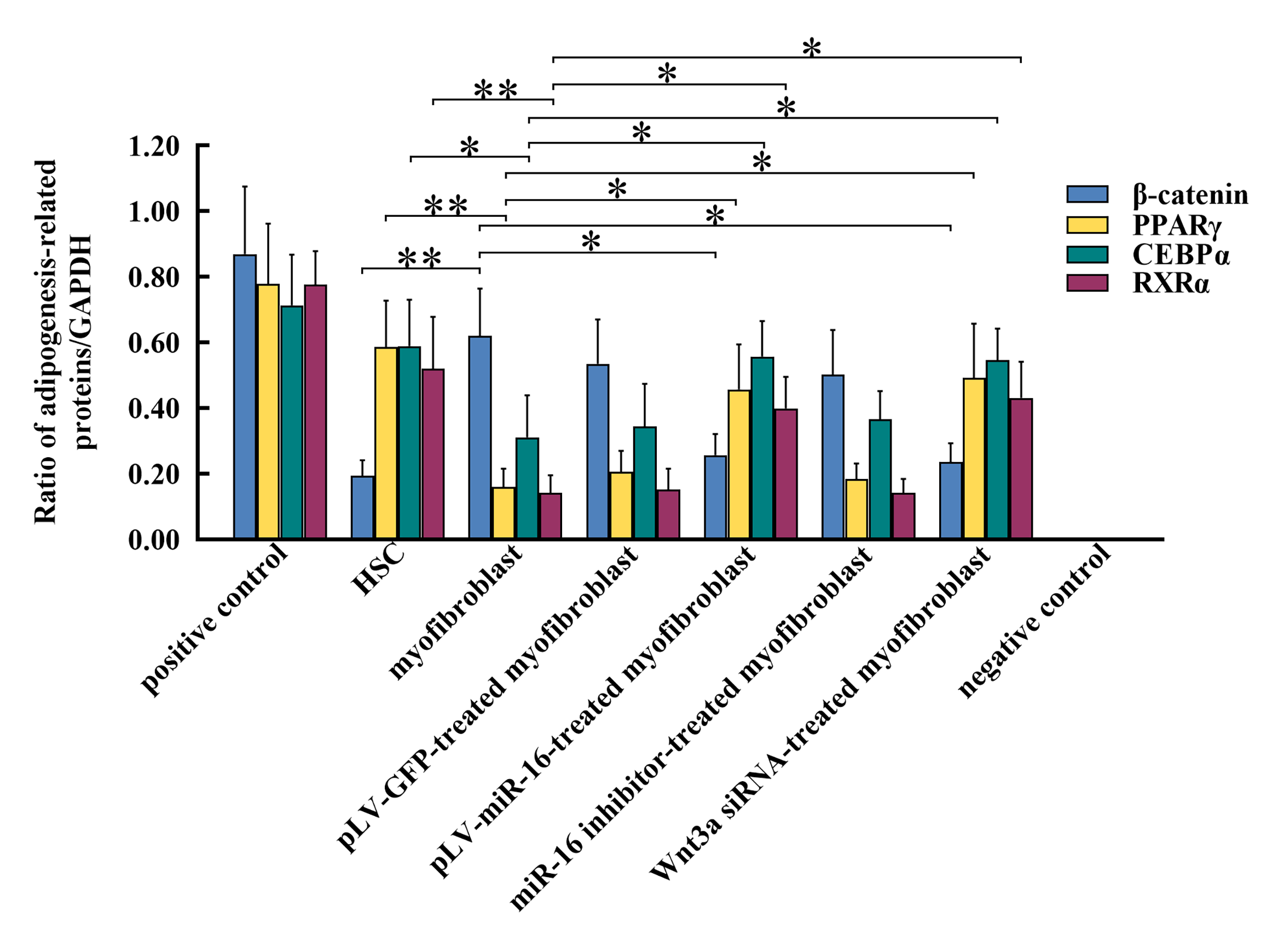

Supplement: Supplementary file 11 — Supplementary Figure 10 [file 41419_2020_2832_MOESM11_ESM.png]

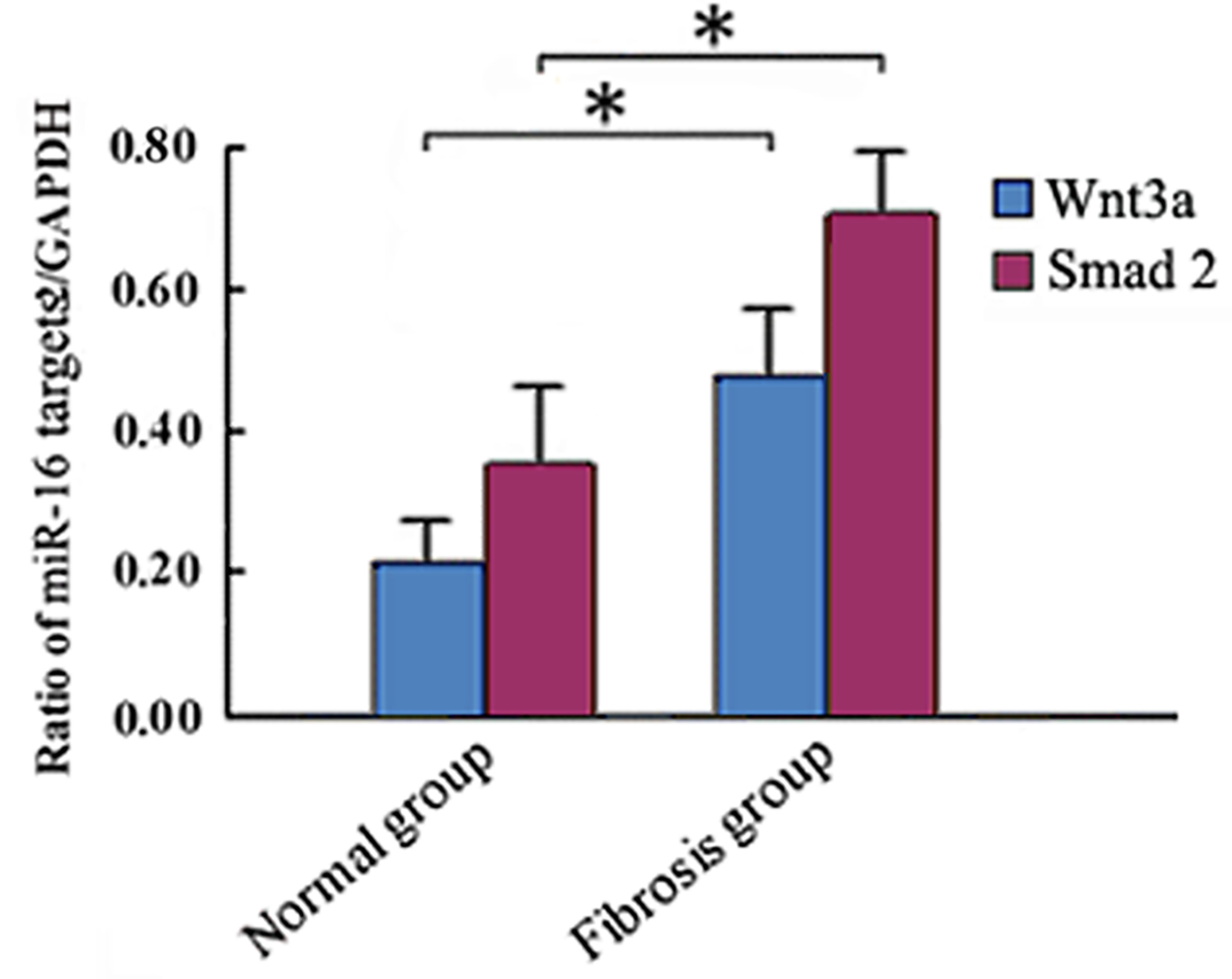

Supplement: Supplementary file 12 — Supplementary Figure 11 [file 41419_2020_2832_MOESM12_ESM.png]

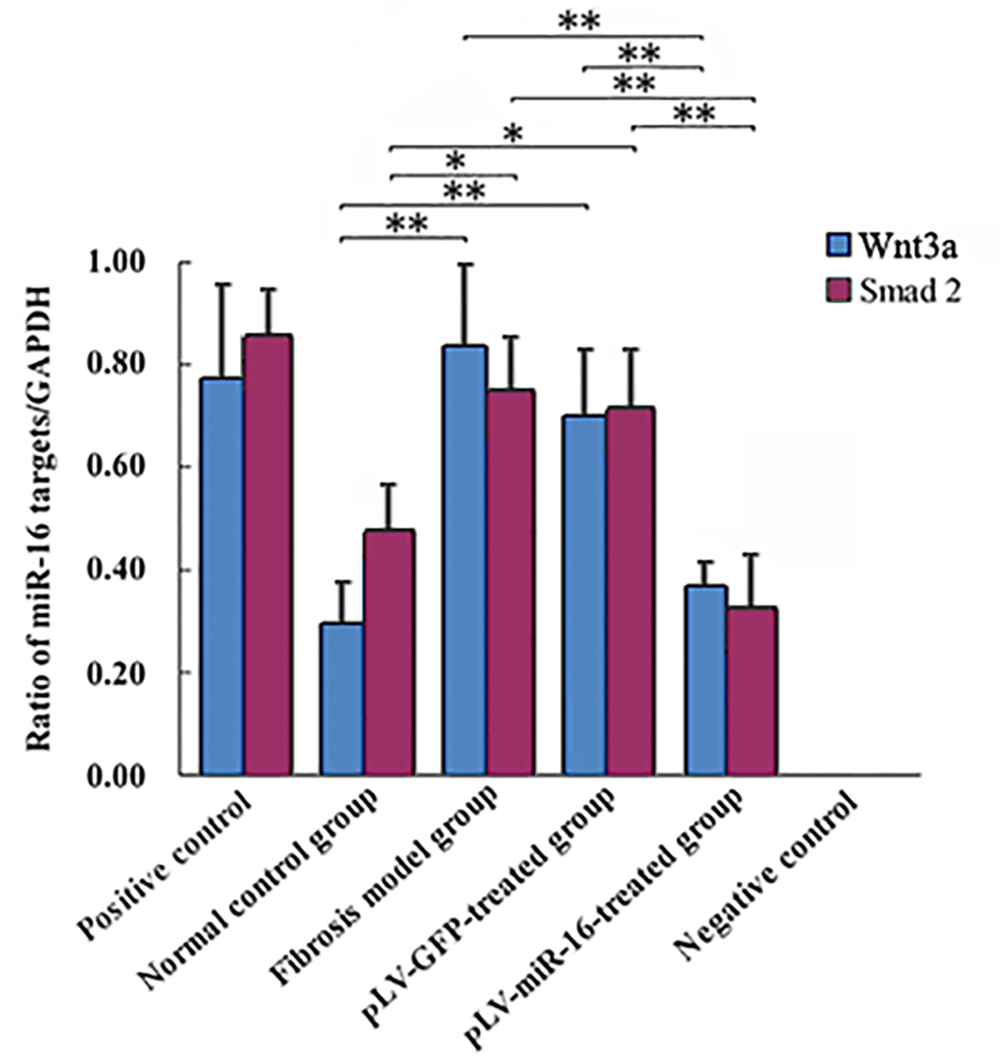

Supplement: Supplementary file 13 — Supplementary Figure 12 [file 41419_2020_2832_MOESM13_ESM.png]

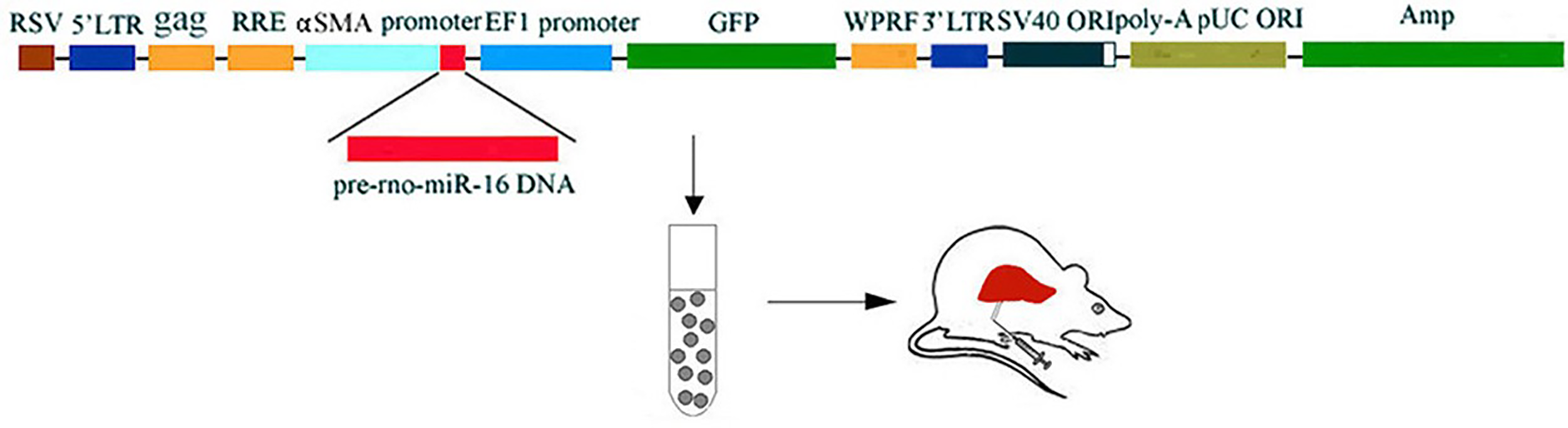

Supplement: Supplementary file 14 — Supplementary Figure 13 [file 41419_2020_2832_MOESM14_ESM.png]

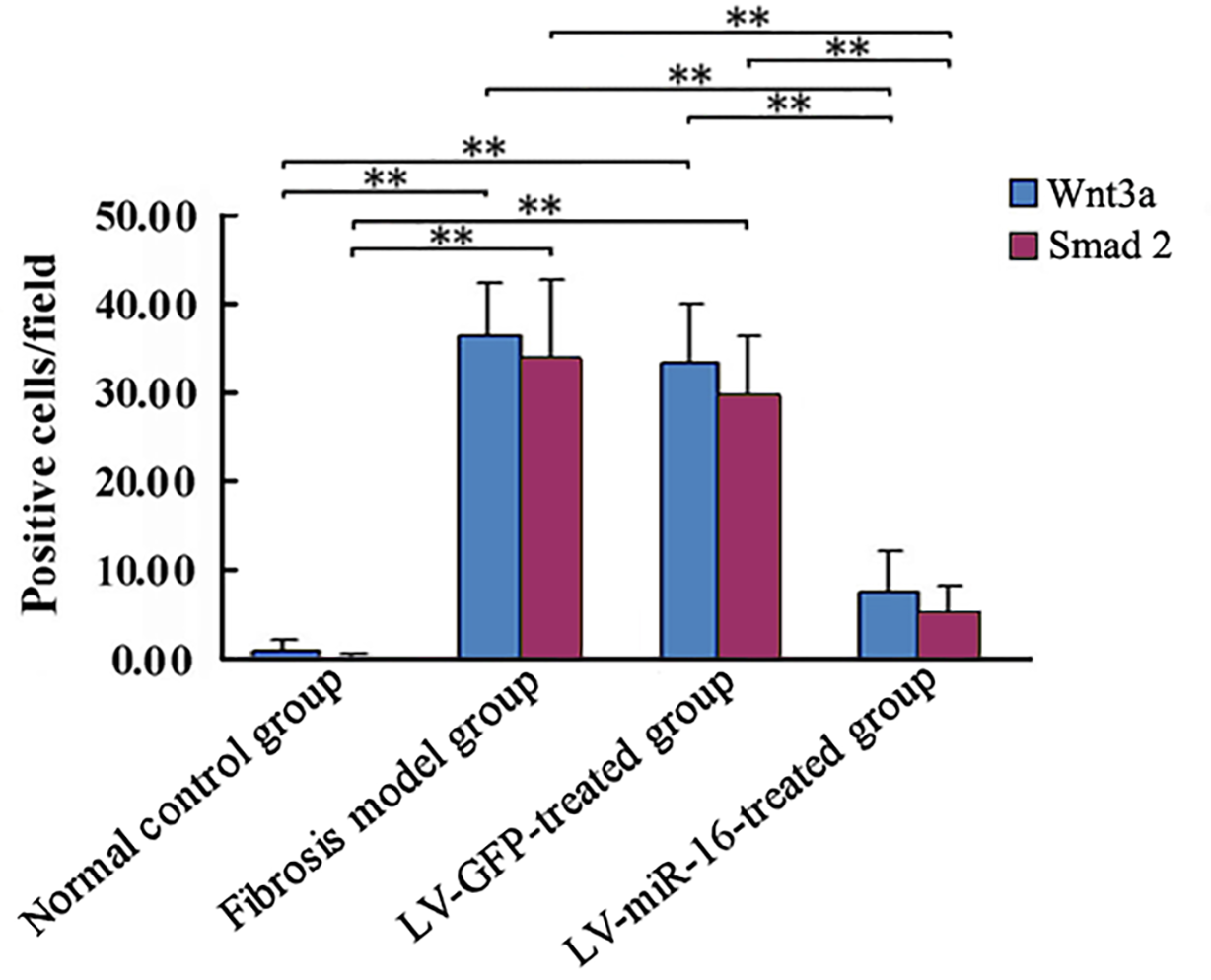

Supplement: Supplementary file 15 — Supplementary Figure 14 [file 41419_2020_2832_MOESM15_ESM.png]

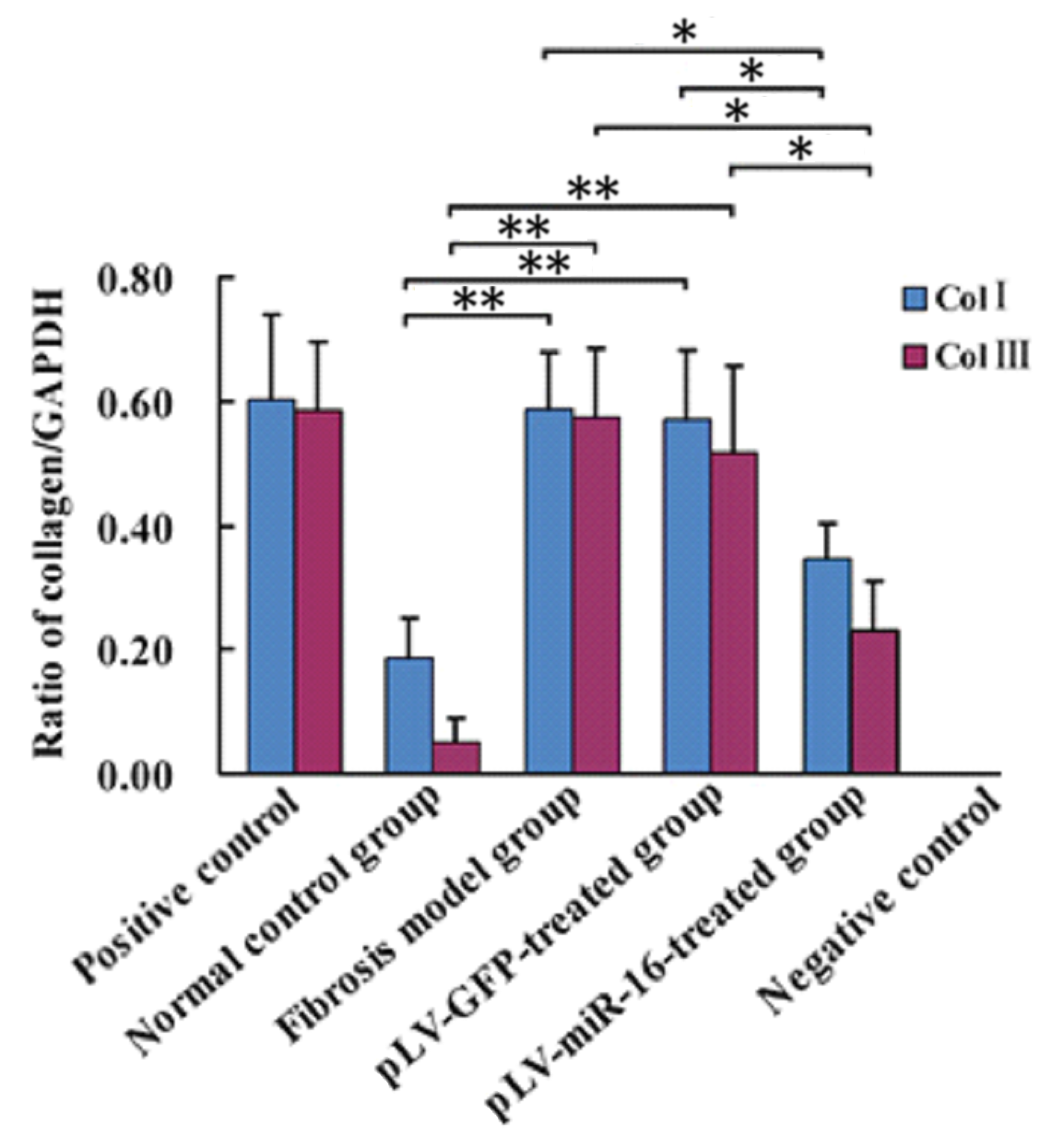

Supplement: Supplementary file 16 — Supplementary Figure 15 [file 41419_2020_2832_MOESM16_ESM.png]

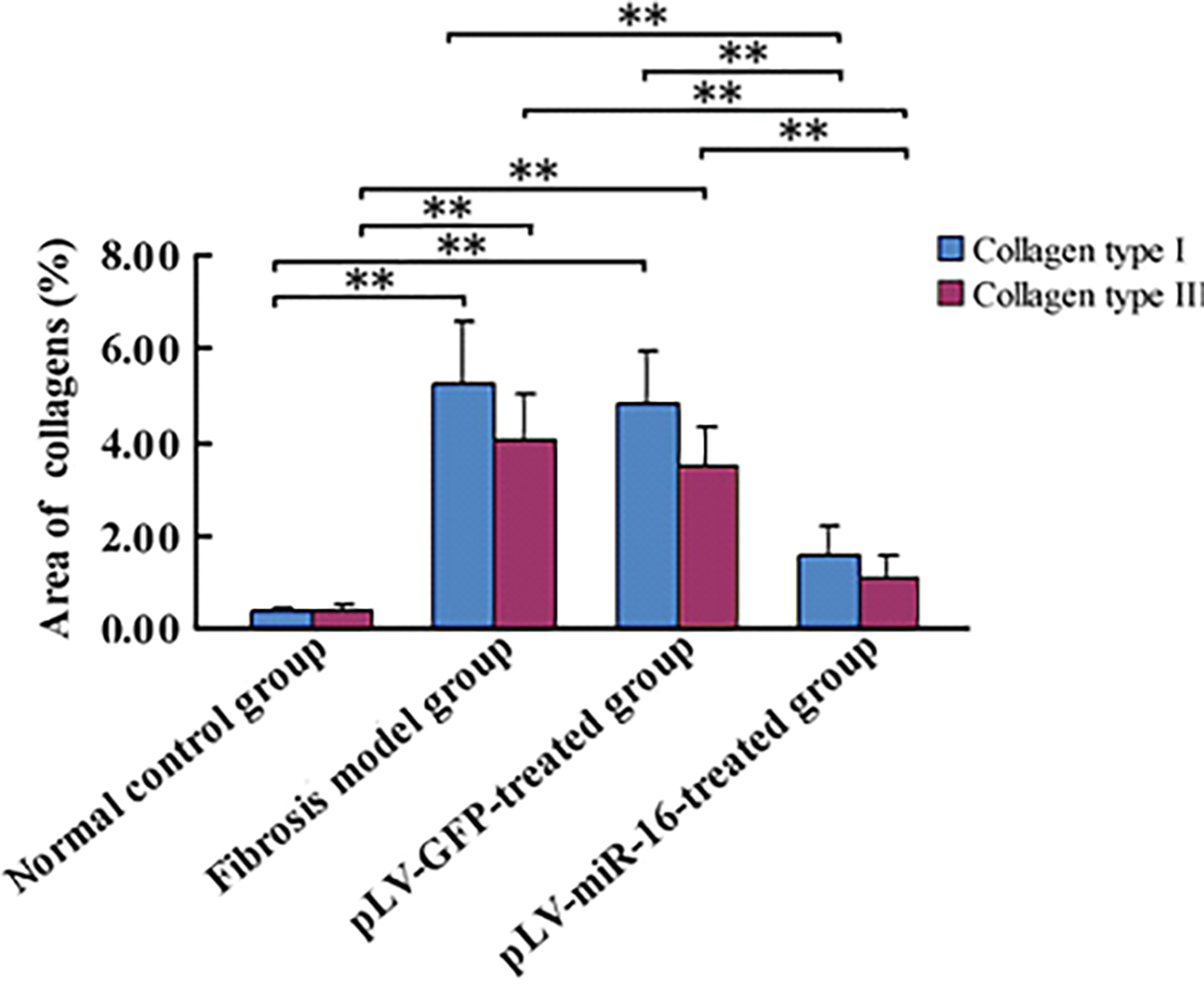

Supplement: Supplementary file 17 — Supplementary Figure 16 [file 41419_2020_2832_MOESM17_ESM.png]
