# Supplementary material for: miR-16 integrates signal pathways in myofibroblasts: determinant of cell fate necessary for fibrosis resolution
Source: Cell Death Dis. 2020 Aug 7;11(8):639. doi: 10.1038/s41419-020-02832-z (PMC7429878; doi:10.1038/s41419-020-02832-z)
Supplement: Supplementary file 18 — Supplementary Table 1 [file 41419_2020_2832_MOESM18_ESM.docx]

**Table S1. Primers used in RT-QPCR**

| **Gene** | **Annealing temperature (℃)** | **Accession No.** | **Product size (bp)** | **Primer (5’-3’)** |
| --- | --- | --- | --- | --- |
| Rat | | | | |
| ALB | 60 | NM_134326 | 321 | F: 5’-GACTGCCCTGTGTGGAAGAC-3’  R: 5’-CGAAGTCACCCATCACCGTC-3’ |
| α-SMA | 59 | NM_031004 | 78 | F: 5’-CGAAGCGCAGAGCAAGAGA-3’  R: 5’-CATGTCGTCCCAGTTGGTGAT-3’ |
| β-catenin | 60 | AF121265.1 | 192 | F: 5’-TGAAGGTGCTGTCTGTCTGC -3’  R: 5’-GCTGCACTAGAGTCCCAAGG -3’ |
| Casp3 | 51 | U49930 | 67 | F:5’-AATTCAAGGGACGGGTCATG-3’  R:5’-GCTTGTGCGCGTACAGTTTC-3’ |
| Cav1 | 61 | BC161826.1 | 150 | F:5’-GCTTCACCACCTTCACTGTGACAA -3’  R:5’-GGAAGCTCTTGATGCACGGTACAA-3’ |
| CD31 | 60 | U77697 | 360 | F:5’-CTTCACCATCCAGAAGGAAGAGAC-3’  R:5’-CACTGGTATTCCATGTCTCTGGTG-3’ |
| CD68 | 60 | NM_001031638 | 62 | F: 5’-TCATGGGAATGCCACAGTTTC-3’  R: 5’-GAGGGCCAACAGTGGAGAA-3’ |
| C/EBPα | 60 | NM_012524.3 | 211 | F: 5’-TGTTGGAGTTGACCAGTGA -3’  R: 5’-ATCCAGCGACCCTAAACCAT-3’ |
| CK19 | 60 | NM_199498 | 388 | F:5’-CACTACGCAGATCCAGATAAACA-3’  R:5’-GAAGTCGCACTGGTAGCAAG-3’ |
| Col1a1 | 61 | Z78279.1 | 245 | F:5’-TCACCTACAGCACGCTTG-3’  R:5’-GGTCTGTTTCCAGGGTTG-3’ |
| Col3a1 | 55 | NM_032085.1 | 175 | F:5’-ATATCAAACACGCAAGGC-3’  R:5’-GATTAAAGCAAGAGGAACAC-3’ |
| Desmin | 56 | NM_022531 | 245 | F: 5’-CTTCAGGAACAGCAGGTC-3’  R: 5’-ATCTCGCAGGTGTAGGAC-3’ |
| Ezr | 51 | NM_019357.1 | 84 | F:5’-AGGTACCGGGCGATGTTCT-3’  R:5’-GGCCTGTTTGGCACTATGTGA-3’ |
| GAPDH | 60 | NM_002046 | 450 | F: 5’-ACCACAGTCCATGCCATCAC-3’  R: 5’-TCCACCACCCTGTTGCTGTA-3’ |
| ICAM1 | 53 | NM_012967 | 252 | F:5’- CCAGACCCTGGAGATGGAGAA -3’  R:5’-AAGCGTCGTTTGTGATCCTCC -3’ |
| Mmp2 | 55 | NM_031054.2 | 119 | F:5’-ACAGGGCAGTGGGATACAGGT-3’  R:5’-AAACAGCAAAGGGCAAACAAAG-3’ |
| PPARγ | 60 | AB011365.1 | 112 | F:5’-TCCGAAGAACCATCCGATTGAA -3’  R:5’-GCAAGGCACTTCTGAAACCGACA -3’ |
| RXRα | 60 | NM_012805.2 | 126 | F:5’-GAGGACATGCCTGTAGAGAAGATT -3’  R:5’-AATGACCCTGTTACCAACATCTGT -3’ |
| SMAD2 | 60 | NM_019191.1 | 104 | F:5’-AACTGCCGCCTCTGGATGAC-3’  R:5’-AGGTGGTGGTGTTTCTGGGAT-3’ |
|  |  |  |  |  |
| **Gene** | **Annealing temperature (℃)** | **Accession No.** | **Product size (bp)** | **Primer (5’-3’)** |
| Wnt3a | 59 | NM_001107005.2 | 172 | F:5’-ATTTGGAGGAATGGTCTCTCG-3’  R:5’-GCAGGTCTTCACTTCGCAAC -3’ |
| Human | | | | |
| Smad2 | 60 | NM_005901.6 | 182 | F:5’-CGTCCATCTTGCCATTCACG-3’  R:5’-CTCAAGCTCATCTAATCGTCCTG-3’ |
| Wnt3a | 60 | NM_033131.4 | 430 | Forward Primer:  5’-AGCTACCCGATCTGGTGGTC-3’  Reverse Primer:  5’-CAAACTCGATGTCCTCGCTAC-3’ |
