# Supplementary material for: miR-16 integrates signal pathways in myofibroblasts: determinant of cell fate necessary for fibrosis resolution
Source: Cell Death Dis. 2020 Aug 7;11(8):639. doi: 10.1038/s41419-020-02832-z (PMC7429878; doi:10.1038/s41419-020-02832-z)
Supplement: Supplementary file 19 — Supplementary Table 2 [file 41419_2020_2832_MOESM19_ESM.docx]

**Table S2. miR-16 regulates transdifferentiation-related genes of myofibroblasts**

| **Gene** | **Abbreviation** | **Accession No.** | **Fold change** | **Signaling pathway** |
| --- | --- | --- | --- | --- |
| **Adipogenesis** | | | | |
| acyl-CoA synthetase long-chain family member 1 | ACSL1 | NM_012820 | 2.28 | Fatty acid metabolism, Adipocytokine signaling pathway, PPAR signaling pathway |
| Adipose differentiation related protein | Adfp | NM_001007144 | 1.94 |  |
| apolipoprotein L 11a | Apol11a | XM_235463 | 2.04 |  |
| CCAAT/enhancer binding protein, α | Cebpa | NM_012524 | 5.73 |  |
| CCAAT/enhancer binding protein, θ | Cebpz | NM_001108701(11) | 1.52 |  |
| elongation of very long chain fatty acids (FEN1/Elo2,SUR4/Elo3, yeast)-like 3 | Elovl3 | NM_001107602 | 9.16 |  |
| elongation of very long chain fatty acids (FEN1/Elo2,SUR4/Elo3, yeast)-like 4 | Elovl4 | XM_001062735(11) | 1.76 |  |
| ELOVL family member 6, elongation of long chain fatty acids | Elovl6 | NM_134383 | -2.00 | Biosynthesis of unsaturated fatty acids |
| fatty acid binding protein 6, ileal | Fabp6 | NM_017098 | 2.58 | PPAR signaling pathway |
| fatty acid binding protein 7, brain | Fabp7 | NM_030832 | 9.43 | PPAR signaling pathway |
| fatty acid desaturase 2 | Fads2 | NM_031344 | -3.14 | Biosynthesis of unsaturated fatty acids, PPAR signaling pathway |
| fatty acid desaturase 3 | Fads3 | NM_173137 | 1.29 |  |
| glycolipid transfer protein domain containing 1 | Gltpd1 | NM_001007703 | 1.77 |  |
| low density lipoprotein receptor adaptor protein 1 | Ldlrap1 | NM_001109271(11) | -1.44 |  |
| **Gene** | **Abbreviation** | **Accession No.** | **Fold change** | **Signaling pathway** |
| oxidized low density lipoprotein (lectin-like) receptor 1 | Olr1 | NM_133306 | 7.87 | PPAR signaling pathway |
| peroxisome proliferator-activated receptor gamma | PPARg | NM_013124 | 1.54 | PPAR signaling pathway |
| retinol binding protein 2, cellular | Rbp2 | NM_012640 | 166.60 |  |
| retinoid X receptor alpha | Rxra | NM_012805 | 4.51 | Adipocytokine signaling pathway, PPAR signaling pathway, Pathways in cancer |
| **Proliferation** | | | | |
| budding uninhibited by benzimidazoles 1 homolog β | Bub1b | XM_001080736 | -1.45 | Cell cycle |
| cyclin D1 | Ccnd1 | NM_171992 | -4.12 | Cell cycle, p53 signaling pathway, Wnt signaling pathway, Focal adhesion, Jak-STAT signaling pathway, Pathways in cancer, Colorectal cancer, Pancreatic cancer, Endometrial cancer, Glioma, Prostate cancer, Thyroid cancer, Melanoma, Bladder cancer, Chronic myeloid leukemia, Acute myeloid leukemia, Small cell lung cancer, Non-small cell lung cancer, Viral myocarditis |
| cyclin M3 | Ccnm3 | NM_001106901 | -1.26 |  |
| cyclin Y | Ccny | XM_001065252 | -1.43 |  |

| **Gene** | **Abbreviation** | **Accession No.** | **Fold change** | **Signaling pathway** |
| --- | --- | --- | --- | --- |
| cell division cycle 20 homolog | Cdc20 | NM_171993 | -1.63 | Cell cycle, Ubiquitin mediated proteolysis |
| cell division cycle 42 (GTP binding protein) | Cdc42 | NM_171994 | -1.18 | Pathways in cancer, Focal adhesion, Adherens junction, Tight junction, MAPK signaling pathway, Chemokine signaling pathway, VEGF signaling pathway, T cell receptor signaling pathway, GnRH signaling pathway, Neurotrophin signaling pathway |
| CDC42 effector protein (Rho GTPase binding) 1 | Cdc42ep1 | NM_00107970 | -3.24 |  |
| CDC42 effector protein (Rho GTPase binding) 2 | Cdc42ep2 | NM_001009689 | -3.43 |  |
| cyclin-dependent kinase inhibitor 1A (p21, Cip1) | Cdkn1a | NM_080782 | 6.63 | Cell cycle, ErbB signaling pathway,  p53 signaling pathway, Pathways in cancer |
| cyclin-dependent kinase inhibitor 1B | Cdkn1b | NM_031762 | -1.78 |  |
| cyclin-dependent kinase inhibitor 2C | Cdkn2c | NM_131902 | -1.68 | Cell cycle |
| CDK5 regulatory subunit associated protein 2 | Cdk5rap2 | XM_001058949 | -1.32 |  |
| fizzy/cell division cycle 20 related 1 | Fzr1 | NM_001108074 | -1.28 | Cell cycle, Ubiquitin mediated proteolysis |
| origin recognition complex, subunit 2-like | Orc2l | NM_001012003 | 1.41 | Cell cycle |
| pituitary tumor-transforming 1 | Pttg1 | NM_022391 | -2.05 | Cell cycle |
| S-phase kinase- associated protein 2 (p45) | Skp2 | NM_001106416 | -1.52 | Cell cycle, Ubiquitin mediated proteolysis, Pathways in cancer |
| ubiquitin-conjugating enzyme E2F | Ube2f | NM_001008381 | -1.23 | Ubiquitin mediated proteolysis |

| **Gene** | **Abbreviation** | | | **Accession No.** | | **Fold change** | **Signaling pathway** | |
| --- | --- | --- | --- | --- | --- | --- | --- | --- |
| wee 1 homolog | | Wee1 | NM_001012742 | | -1.77 | | | Cell cycle |
| **ECM metabolism** | | | | | | | | |
| collagen, type I, α1 | Col1a1 | | | NM_053304 | | -2.72 | Focal adhesion | |
| collagen, type III, α1 | Col3a1 | | | NM_032085 | | -14.83 | Focal adhesion | |
| collagen, type IV, α 5 | Col4a5 | | | XM_343778 | | -2.75 |  | |
| collagen, type XII, α1 | Col12a1 | | | XM_00106068 | | -2.33 |  | |
| collagen, type XIV, α1 | Col14a1 | | | NM_001130548(11) | | -3.92 |  | |
| collagen, type XVI, α1 | Col16a1 | | | NM_001015033 | | -1.86 |  | |
| collagen, type XVII, α1 | Col17a1 | | | NM_001106366 | | 1.27 |  | |
| collagen, type VIII, α1 | Col8a1 | | | NM_001107100 | | -2.70 |  | |
| fibronectin 1 | Fn1 | | | NM_019143 | | -1.27 | Focal adhesion, Pathways in cance | |
| laminin, α2 | Lama2 | | | XM_001062273 | | -1.68 | Focal adhesion, Pathways in cancer | |
| latent transforming growth factor β binding protein 4 | Ltbp4 | | | XM_001077795 | | -3.00 | TGF-β signaling pathway, Cell cycle, MAPK signaling pathway, Pathways in cancer | |
| matrix metallopeptidase 2 | Mmp2 | | | NM_03105 | | 3.82 | Pathways in cancer, GnRH signaling pathway | |
| procollagen-proline, 2-oxoglutarate 4-dioxygenase (proline 4-hydroxylase) α | P4ha3 | | | XM_001066817(11) | | -2.53 |  | |
| Transforming growth factor β2 | Tgfb2 | | | NM_031131 | | -2.54 |  | |
| SMAD family member 2 | Smad2 | | | NM_019191 | | -1.64 | Cell cycle, Wnt signaling pathway, TGF-beta signaling pathway, Adherens junction, Pathways in cancer, Colorectal cancer, Pancreatic cancer, | |
| **Apoptosis** | | | | | | | | |
| BCL2-like 13 (apoptosis facilitator) | | Bcl2l13 | NM_001107885 | | 1.27 | | |  |

| **Gene** | **Abbreviation** | **Accession No.** | **Fold change** | **Signaling pathway** |
| --- | --- | --- | --- | --- |
| baculoviral IAP repeat-containing 2 | Birc2 | NM_021752 | 1.43 | Apoptosis, Ubiquitin mediated proteolysis, Focal adhesion, NOD-like receptor signaling pathway, Pathways in cancer |
| baculoviral IAP repeat-containing 3 | Birc3 | NM_023987 | 2.41 | Apoptosis, Ubiquitin mediated proteolysis, Focal adhesion, NOD-like receptor signaling pathway, Pathways in cancer |
| calpain 1 | Capn1 | NM_019152 | -1.49 | Apoptosis |
| caspase 3 | Casp3 | NM_012922 | 1.75 | Apoptosis, p53 signaling pathway, MAPK signaling pathway, Pathways in cancer |
| caspase 9 | Casp9 | NM_031632 | 4.85 | Apoptosis p53 signaling pathway, Pathways in cancer, VEGF signaling pathway |
| DNA-damage inducible transcript 3 | Ddit3 | NM_024134 | 4.45 | MAPK signaling pathway |
| DNA fragmentation factor, α subunit | Dffa | NM_053679 | 1.59 | Apoptosis |
| growth arrest and DNA-damage-inducible, γ | Gadd45g | NM_001077640 | 2.92 | Cell cycle, p53 signaling pathway, MAPK signaling pathway |
| PRKC, apoptosis, WT1, regulator | Pawr | NM_033485 | -1.47 |  |

| **Gene** | **Abbreviation** | **Accession No.** | **Fold change** | **Signaling pathway** |
| --- | --- | --- | --- | --- |
| protein phosphatase 3, catalytic subunit, β isoform | Ppp3cb | NM_017042 | -1.38 | Apoptosis, Wnt signaling pathway, MAPK signaling pathway, Calcium signaling pathway, VEGF signaling pathway, T cell receptor signaling pathway, B cell receptor signaling pathway |
| protein kinase, cAMP dependent regulatory, type I, α | Prkar1a | NM_013181 | -1.18 | Apoptosis, Insulin signaling pathway |
| serine (or cysteine) peptidase inhibitor, clade E, member 1 | Serpine1 | NM_012620 | 4.29 | p53 signaling pathway |
| seven in absentia 1A | Siah1a | NM_080905 | -1.50 | p53 signaling pathway, Wnt signaling pathway, Ubiquitin mediated proteolysis |
| transmembrane BAX inhibitor motif containing 6 | Tmbim6 | NM_019381 | 1.29 |  |
| tumor protein p53 inducible nuclear protein 1 | Tp53inp1 | NM_181084 | 2.55 |  |
