# Supplementary material for: miR-16 integrates signal pathways in myofibroblasts: determinant of cell fate necessary for fibrosis resolution
Source: Cell Death Dis. 2020 Aug 7;11(8):639. doi: 10.1038/s41419-020-02832-z (PMC7429878; doi:10.1038/s41419-020-02832-z)
Supplement: Supplementary file 20 — Supplementary Table 3 [file 41419_2020_2832_MOESM20_ESM.docx]

**Table S3. miR-16 regulates transdifferentiation-related GOs of myofibroblasts**

| **GO** | **Term** | ***P* Value** | **Fold**  **Enrichment** | FDR |
| --- | --- | --- | --- | --- |
| 0007242 | intracellular signaling cascade | 7.52 E-04 | 1.49 | 1.35 |
| 0010033 | response to organic substance | 1.30 E-03 | 1.46 | 2.31 |
| 0042981 | regulation of apoptosis | 1.61 E-05 | 1.77 | 0.029 |
| 0043067 | regulation of programmed cell death | 2.42 E-05 | 1.75 | 0.044 |
| 0010941 | regulation of cell death | 2.74 E-05 | 1.74 | 0.049 |
| 0008104 | protein localization | 3.21 E-04 | 1.64 | 0.58 |
| 0006350 | transcription | 1.38 E-03 | 1.55 | 2.46 |
| 0042127 | regulation of cell proliferation | 2.09 E-03 | 1.53 | 3.70 |
| 0033554 | cellular response to stress | 1.33 E-07 | 2.25 | 2.39 E-04 |
| 0015031 | protein transport | 8.29 E-05 | 1.79 | 0.15 |
| 0045184 | establishment of protein localization | 1.04 E-04 | 1.77 | 0.19 |
| 0043933 | macromolecular complex subunit organization | 2.66 E-03 | 1.57 | 4.68 |
| 0046907 | intracellular transport | 1.85 E-04 | 1.80 | 0.33 |
| 0065003 | macromolecular complex assembly | 2.60 E-03 | 1.59 | 4.58 |
| 0007049 | cell cycle | 1.63 E-04 | 1.83 | 0.29 |
| 0009611 | response to wounding | 1.17 E-04 | 1.90 | 0.21 |
| 0022610 | biological adhesion | 7.09 E-04 | 1.74 | 1.27 |
| 0007155 | cell adhesion | 7.09 E-04 | 1.74 | 1.27 |
| 0043066 | negative regulation of apoptosis | 2.44 E-06 | 2.30 | 4.40 E-03 |
| 0043069 | negative regulation of programmed cell death | 3.52 E-06 | 2.26 | 6.34 E-03 |
| 0060548 | negative regulation of cell death | 3.74 E-06 | 2.26 | 6.74 E-03 |
| 0008219 | cell death | 2.86 E-04 | 1.85 | 0.51 |
| 0016265 | death | 3.99 E-04 | 1.82 | 0.72 |
| 0019725 | cellular homeostasis | 1.64 E-03 | 1.69 | 2.91 |
| 0012501 | programmed cell death | 3.95 E-04 | 1.88 | 0.71 |
| 0042325 | regulation of phosphorylation | 1.16 E-03 | 1.77 | 2.07 |
| **GO** | **Term** | ***P* Value** | **Fold**  **Enrichment** | FDR |
| 0051174 | regulation of phosphorus metabolic process | 2.26 E-03 | 1.70 | 3.99 |
| 0019220 | regulation of phosphate metabolic process | 2.26 E-03 | 1.70 | 3.99 |
| 0006915 | apoptosis | 1.10 E-03 | 1.81 | 1.96 |
| 0055082 | cellular chemical homeostasis | 1.51 E-03 | 1.78 | 2.68 |
